# Reversible Hydride Migration from C_5_Me_5_ to Rh^I^ Revealed by a Cooperative Bimetallic Approach

**DOI:** 10.1002/anie.202008442

**Published:** 2020-09-09

**Authors:** Macarena G. Alférez, Juan J. Moreno, Nereida Hidalgo, Jesús Campos

**Affiliations:** ^1^ Instituto de Investigaciones Químicas (IIQ) Departamento de Química Inorgánica and Centro de Innovación en Química Avanzada (ORFEO-CINQA) Consejo Superior de Investigaciones Científicas (CSIC) University of Sevilla Avenida Américo Vespucio 49 41092 Sevilla Spain

**Keywords:** cyclopentadienyl ligands, hydride migration, metal–ligand cooperation, non-innocent ligands, transition metals

## Abstract

The use of cyclopentadienyl ligands in organometallic chemistry and catalysis is ubiquitous, mostly due to their robust spectator role. Nonetheless, increasing examples of non‐innocent behaviour are being documented. Here, we provide evidence for reversible intramolecular C−H activation at one methyl terminus of C_5_Me_5_ in [(*η*‐C_5_Me_5_)Rh(PMe_3_)_2_] to form a new Rh−H bond, a process so far restricted to early transition metals. Experimental evidence was acquired from bimetallic rhodium/gold structures in which the gold center binds either to the rhodium atom or to the activated Cp* ring. Reversibility of the C−H activation event regenerates the Rh^I^ and Au^I^ monometallic precursors, whose cooperative reactivity towards polar E−H bonds (E=O, N), including the N−H bonds in ammonia, can be understood in terms of bimetallic frustration.

## Graphical abstract

The reversible migration of hydrogen atoms from methyl groups of the ubiquitous C_5_Me_5_ (Cp*) ligand to the Rh^I^ center in [(η^5^‐C_5_Me_5_)Rh(PMe_3_)_2_] is demonstrated, revealing the potential role of Cp* as a proton shuttle by a route that was previously restricted to early transition metals. The incorporation of bulky and electrophilic gold fragments allows the isolation of key bimetallic species in support of the reversible C−H activation at Cp*.

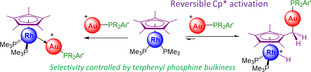

Cyclopentadienyl ligands (C_5_R_5_^−^) are undoubtedly among the most widely used stabilizing fragments in organometallic chemistry and homogeneous catalysis.[^1^, ^2^] They exhibit variable hapticity (ranging from *η* ^1^ to *η* ^5^)^[3]^ and have a rich capacity to bind, in essence, any metallic element across the periodic table.^[4]^ Their ubiquity is largely related to their reliability as robust spectator ligands. Nonetheless, there is increased interest in their utility as non‐innocent motifs. This is propelled by rapid progress in the broad field of cooperative chemistry.^[5]^ For example, protonation of (C_5_R_5_)M fragments can take place either at the metal center^[6]^ or on the Cp ring,^[7]^ with formal proton migration^[8]^ permitting the two structures to interconvert (Figure 1 A).^[9]^ This has been recently exploited^[10]^ to design proton‐coupled‐electron‐transfer (PCET) processes applied to hydrogen evolution^[11]^ or dinitrogen reduction^[12]^ reactions.

**Figure 1:**
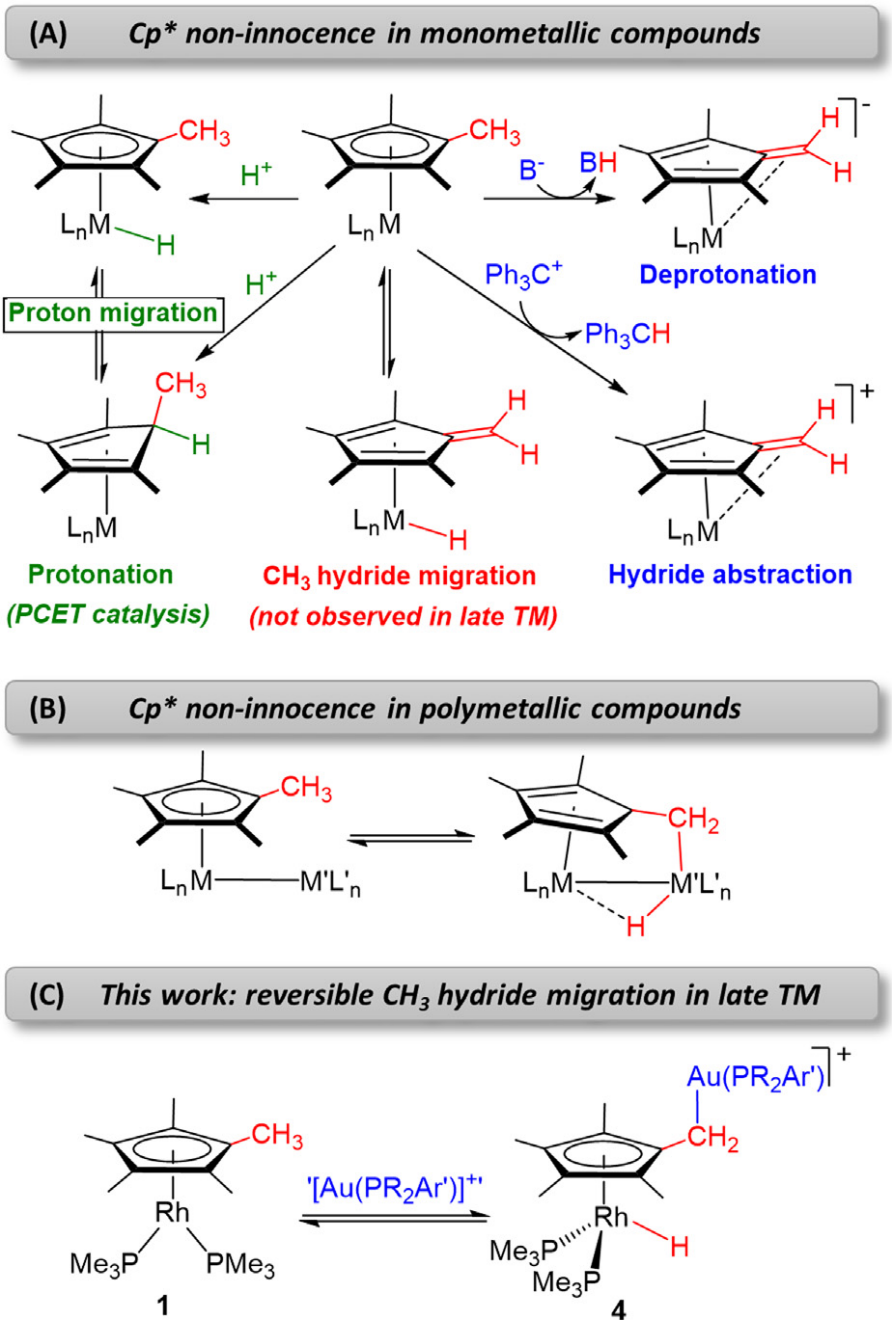
Previous routes for the activation of Cp* (and related) ligands in A) mononuclear compounds, B) polymetallic species and C) this work, where hydride migration to a rhodium center is promoted by electrophilic gold fragments (PCET=proton‐coupled electron transfer; M=transition metal).

Permethylated cyclopentadienyl ligands, particularly C_5_Me_5_ (Cp*), enable additional activation at the CH_3_ units (Figure 1 A). Deprotonation involving a redox event at the metal by treatment with an external base or through an intramolecular pathway by means of a basic anchoring ligand is well documented,^[13]^ forming tuck‐in complexes^[14]^ and, in some cases, leading to stable fulvene‐containing structures.^[15]^ Hydride abstraction has also been demonstrated, proceeding in two steps through one‐electron oxidation followed by hydrogen atom abstraction.^[16]^ Direct hydride migration from a methyl group to the metal was soon recognized for early transition metals,^[17]^ but to the best of our knowledge there is still no evidence for this with late transition metal analogues.^[18]^ Alternatively, the incorporation of a second metal center in close proximity (i.e. in cluster structures) allows for C−H bond activation of one methyl terminus (Figure 1 B) to form tuck‐over complexes,^[19]^ with reversibility being documented in some cases.^[20]^

In the context of bimetallic systems and driven by our search for metal‐only frustrated Lewis pairs,^[21]^ we report here the intriguing activation of one of the methyl groups of the C_5_Me_5_ ligand in [(*η* ^5^‐C_5_Me_5_)Rh(PMe_3_)_2_] (**1**).^[22]^ We anticipated that bimetallic frustration could be achieved by using sterically congested and electrophilic gold fragments. With this in mind, we have explored the reactivity of **1** with gold(I) compounds stabilized by bulky terphenyl phosphine ligands and bearing a weakly coordinating triflimide anion, [(PR_2_Ar′)Au(NTf_2_)] (R=Me, Cyp; Ar′=C_6_H_3_‐2,6‐Ar_2_; NTf_2_=[N(SO_2_CF_3_)_2_]^−^) (**2**).^[23]^ Rather than the predicted frustration, the most hindered gold species promote hydride migration within the rhodium fragment (Figure 1 C). These results provide the first unambiguous demonstration for reversible hydride migration from a methyl group of C_5_Me_5_ to a late transition metal, highlighting the potential for the development of PCET‐based catalytic cycles involving CH_3_ groups as proton shuttles.^[24]^

Evidence for Cp* activation at **1** is dependent on the shielding capacity of the terphenyl phosphine that binds the Au^I^ fragment. We have examined three ligands whose steric profile follows the order PCyp_2_Ar^Xyl2^ > PMe_2_Ar^Tripp2^ > PMe_2_Ar^Xyl2^ (Cyp=C_5_H_10_; see Scheme 1). Treatment of **1** with [(PMe_2_Ar^Xyl2^)Au(NTf_2_)] (**2 a**) readily caused quantitative formation of the new metal‐only Lewis pair (MOLP)^[25]^ **3 a** (Scheme 1). This was identified by a new AB_2_ pattern in the ^31^P{^1^H} NMR spectrum, where an apparent double triplet at 13.9 ppm (^3^ *J*_PP_=12, ^2^ *J*_PRh_=10 Hz) and a double doublet at −3.1 ppm (^3^ *J*_PP_=12, ^1^ *J*_PRh_=155 Hz) due to PMe_2_Ar^Xyl2^ and the two PMe_3_ ligands, respectively, are evidence for the formation of a bimetallic species.

**Scheme 1:**
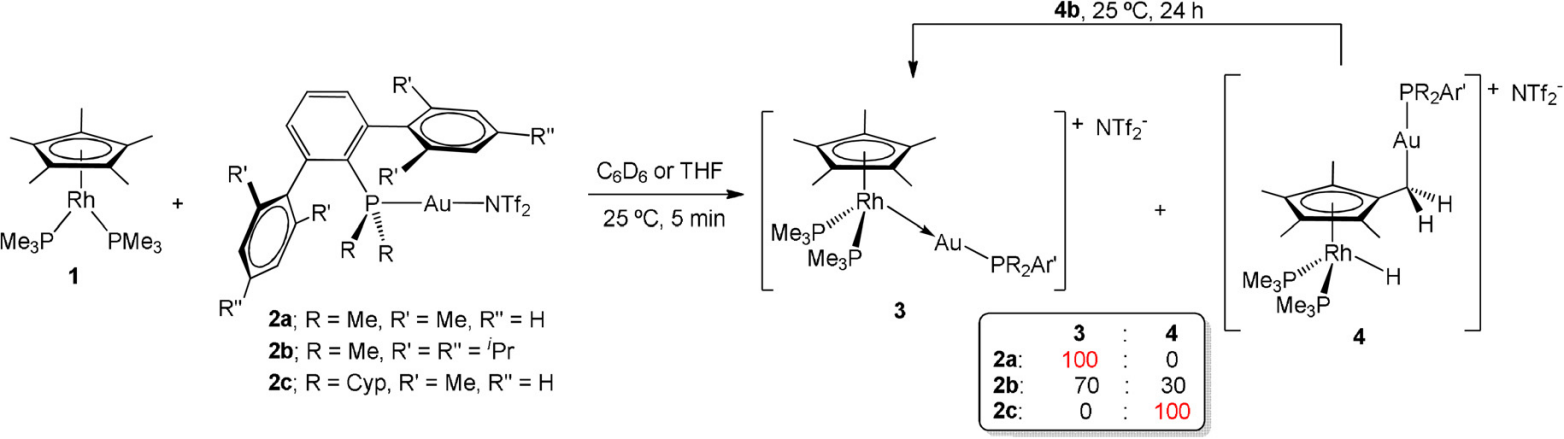
Selectivity in the reaction between Rh^I^ compound **1** and electrophilic Au^I^ species of type **2**.

In stark contrast, addition of [(PCyp_2_Ar^Xyl2^)Au(NTf_2_)] (**2 c**) to benzene or THF solutions of **1** immediately led to compound **4 c**, which formed in quantitative spectroscopic yields (Scheme 1). ^1^H NMR analysis revealed the asymmetry of the cyclopentadienyl ring which, instead of the usual large singlet, appeared as three resonances: two at 1.84 and 1.73 ppm for six protons each, and a doublet at 1.05 ppm (^2^ *J*_HP_=9.6 Hz) pertaining to the functionalized C_5_Me_4_‐*CH_2_*‐Au moiety. These were accompanied by a distinctive low‐frequency signal at −13.34 ppm (dt, ^2^ *J*_HP_=35.8, ^1^ *J*_HRh_=24.5 Hz) due to a newly formed hydride ligand bound to the rhodium center. As supported by isotopic labelling experiments (vide infra), this hydride originates from a methyl group of Cp*, showing evidence that this type of hydrogen shuttle is viable in late transition metals. The absence of ^31^P‐^103^Rh scalar coupling for the terphenyl phosphine, which resonates at 56.0 (t, ^5^ *J*_PP_=10 Hz) is characteristic of compounds **4**, while PMe_3_ ligands in **4 c** display a signal at −2.2 ppm (dd, ^5^ *J*_PP_=10, ^1^ *J*_PRh_=140 Hz).

Interestingly, we noted an in‐between scenario with the intermediate size phosphine (PMe_2_Ar^Tripp2^) of compound **2 b**. Addition of [(PMe_2_Ar^Tripp2^)Au(NTf_2_)] (**2 b**) to solutions of **1** results in the formation of both isomers, **3 b** and **4 b**, in a ca. 70:30 ratio, along with unidentified minor species. Compounds **3 b** and **4 b** have the same spectroscopic pattern as their PMe_2_Ar^Xyl2^ and PCyp_2_Ar^Xyl2^ analogues (Figure S1), which allowed us to easily assign their molecular formulation. That is, while the bimetallic adduct **3 b** exhibits ^31^P{^1^H} resonances at 15.2 (dt, ^3^ *J*_PP_=14, ^2^ *J*_PRh_=12 Hz, PMe_2_Ar^Tripp2^) and −5.1 (dd, ^3^ *J*_PP_=14, ^1^ *J*_PRh_=155 Hz, PMe_3_)  ppm, isomer **4 b** displays signals at 12.7 (t, ^3^ *J*_PP_=12 Hz, PMe_2_Ar^Tripp2^) and −2.2 (dd, ^3^ *J*_PP_=12 Hz, ^1^ *J*_PRh_=140 Hz, PMe_3_), as well as a characteristic hydride peak at −13.48 (^2^ *J*_HP_=35.8, ^1^ *J*_HRh_=24.1 Hz,) ppm. Low‐temperature NMR monitoring of the reaction revealed that isomer **3 b** forms as the exclusive product below −40 °C, while the complex derived from C−H activation at the Cp* moiety requires higher temperatures to appear. Nevertheless, heating solutions of pure **3 b** did not cause the appearance of isomer **4 b**. We also observed that the stability of compounds **3** and **4** clearly differs. While the former remain intact for weeks in solution under a nitrogen atmosphere, the latter complexes slowly decompose under the same conditions. In the case of **4 b**, we were thrilled to note that at least part of this species evolves to its isomeric bimetallic adduct **3 b**, thus supporting the reversibility for the Cp* activation process. Further evidence for this will be discussed later.

We authenticated the molecular structures of **3 b** and **4 c** by X‐ray diffraction studies (Figure 2), for which single crystals were grown by slow diffusion of pentane into their benzene solutions. As expected, the rhodium center in the two structures adopts a piano‐stool conformation. Compound **3 b** exhibits a Rh‐Au bond distance of 2.593(1) Å, comparable to the only other example of a bimetallic Rh/Au unsupported MOLP of this kind.^[26]^ This value is shortened by around 0.2 Å with respect to the sum of their covalent radii (2.78 Å),^[27]^ thus providing support for the existence of a dative Rh→Au bond. Similarly, the calculated formal shortness ratio (FSR),^[28]^ defined as the ratio between the M−M length and the sum of metallic radii, exactly accounts for 1.00. The structure of **4 c** finds no previous precedent. In view of the isolobal analogy between H^+^ and [LAu]^+^ fragments, it could be described as the substitution of a methyl proton of the Cp* ligand by the [(PCyp_2_Ar^Xyl2^)Au]^+^ unit, which is accommodated in an orthogonal fashion relative to the Cp* plane (85.21°). The highly electrophilic character of gold(I) is likely responsible for shortening the C1−C6 distance to 1.483(10) Å (*c.f*. average 1.51 Å for *d* ${}_{\left(\mathrm{Cp}*\right)C\text{-}\mathrm{CH}{}_{3}}$ ).

**Figure 2:**
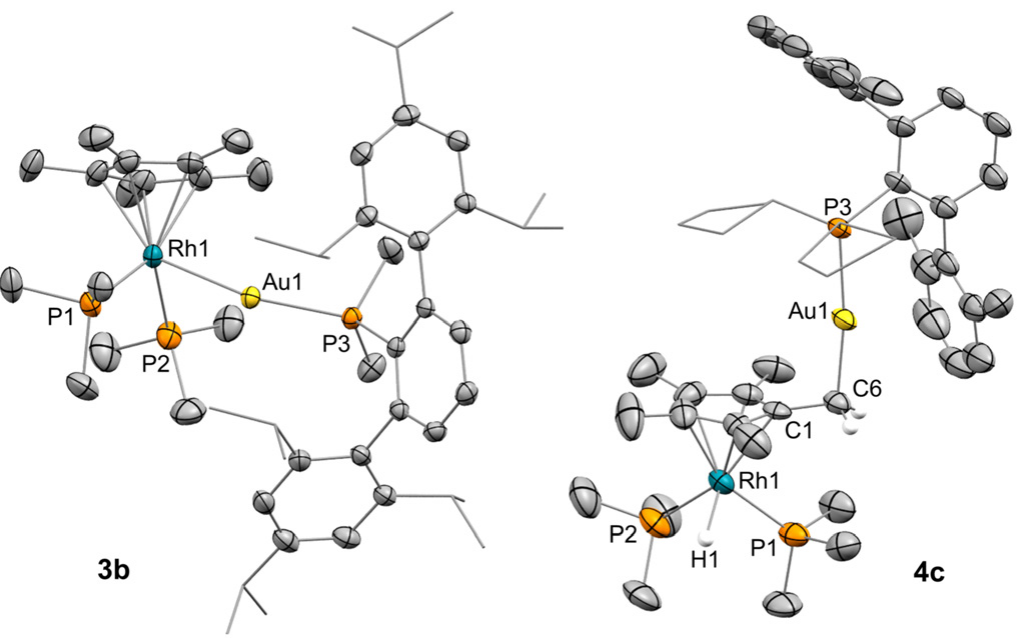
ORTEP diagram of compounds **3 b** and **4 c**; for the sake of clarity most hydrogen atoms, as well as solvent molecules and triflimide counteranions are excluded, while some fragments are represented in wireframe format and thermal ellipsoids are set at 50 % probability.

As introduced above, there is no previous demonstration for the migration of a hydride from a methyl group of Cp* to a late transition metal, although the possibility has been invoked before.^[18]^ In order to demonstrate the origin of the hydride ligand in compounds **4** we aimed to access an perdeuterated isotopologue of **1**. The cyclopentadienyl ligand in **1** was fully labelled by heating in CD_3_OD solution at 90 °C for 30 hours without any apparent decomposition (Scheme 2 a; Figure S3). Full deuteration of Cp* is well documented; however, it typically requires either the addition of an external base to favour deprotonation/protonation routes or the presence of a basic ligand for the same purpose.[^18a^, ^29^] The deuteration of **1** in the absence of external additives speaks in favour of the basic role played by the rhodium center. Brookhart has suggested that deuteration of related [(*η* ^5^‐C_5_Me_5_)Rh(olefin)_2_] compounds may proceed through a bimolecular route,^[18d]^ though it does not seem to apply in this case, where the disappearance of the ^1^H NMR signal due to Cp* follows first‐order kinetics (*k*_1_=6.66×10^−5^ s^−1^; Figure S4). As anticipated, treatment of isotopologue **1‐*d***_**15**_ with [(PCyp_2_Ar^Xyl2^)Au(NTf_2_) (**2 c**) immediately yields the corresponding bimetallic product derived from Cp* activation, for which the absence of a low‐frequency ^1^H NMR signal illustrates the migration of the deuterium from a methyl group of the Cp* ring.

**Scheme 2:**
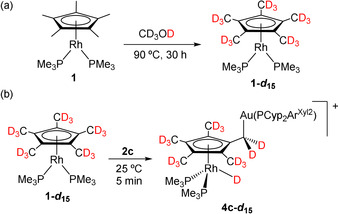
a) Isotopic labelling of **1** in CD_3_OD and b) synthesis of isotopologue **4 c**,***d***_**15**_ by reaction of **1‐*d***_**15**_ and **2 c**.

We gained further evidence for the reversibility of the Rh/CH_3_ hydride migration process by conducting reactivity studies and taking advantage of additional isotopic labelling experiments. We explored the reactivity of Rh/Au bimetallic compounds **3** and **4** towards polar E−H bonds, particularly those of NH_3_, MeOH and H_2_O. Their activation by transition metal complexes is often challenging, particularly for the case of the N−H bonds in ammonia.^[30]^ This is due to the formation of Werner‐type complexes that typically quench any further reactivity at the metal site. There are, however, examples that demonstrate the potential of bimetallic entities to tackle these difficulties.^[31]^ In the present case, we noticed that metal adducts **3** exhibit no reactivity towards the explored E−H bonds, while compounds of type **4** were active even under very mild conditions (Scheme 3 a), particularly for N‐H activation. This behaviour was especially obvious for the mixture of **3 b** and **4 b**, where only the Cp*‐activated compound (**4 b**) evolved and the bimetallic adduct (**3 b**) remained unaltered (Figure S2).

**Scheme 3:**
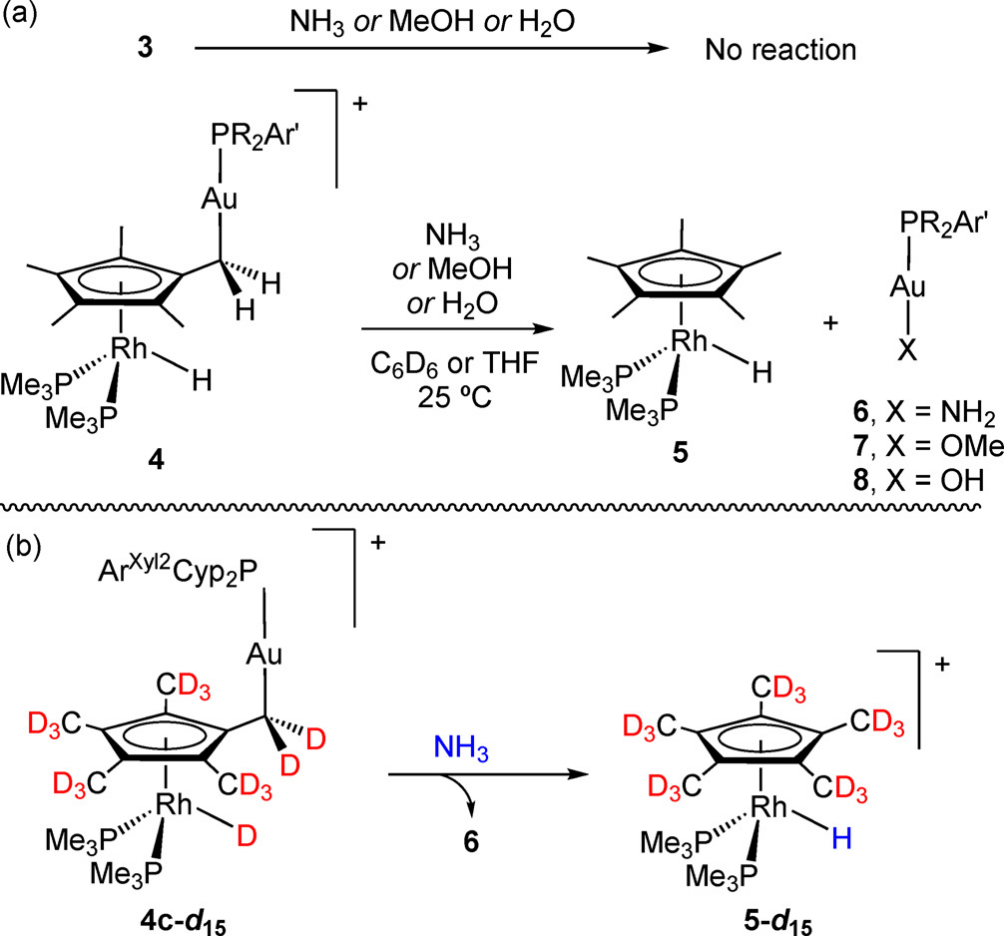
a) Reactivity studies of bimetallic Rh/Au compounds towards E−H bonds of ammonia, methanol and water and b) reaction between isotopically labelled **4 c**,***d***_***15***_ and ammonia.

For convenience, we focused on the reactivity of **4 c** to inspect these reactions in more detail. This compound evolved into equimolar mixtures of cation [(*η* ^5^‐C_5_Me_5_)Rh(PMe_3_)_2_H]^+^ (**5**)^[22]^ and the corresponding neutral gold complex [(PCyp_2_Ar^Xyl2^)Au(X)] (where X stands for NH_2_ (**6**), OMe (**7**) or OH (**8**); Scheme 3 a). To confirm the nature of the gold(I) species, these were independently prepared by conventional methods (see SI for details). The rate of E−H bond activation varied markedly for the three substrates. While the N−H bond in ammonia was readily cleaved (*t*_1/2_≈30 min), the activation of methanol and water was significantly slower (MeOH: *t*_1/2_≈48 h; H_2_O: *t*_1/2_≈5 days). We hypothesized that the reduced activity of **4 c** towards methanol and water is likely derived from the low oxophilicity of gold,^[32]^ and in the latter case because of solubility issues. Importantly, when the reaction with ammonia was carried out using labelled **4 c**,***d***_**15**_, a distinctive hydridic signal became discernible by ^1^H NMR spectroscopy (Scheme 3 b). Similarly, the reaction of non‐deuterated **4 c** with CD_3_OD yields [(*η* ^5^‐C_5_Me_5_)Rh(PMe_3_)_2_D]^+^ as the major Rh‐containing species. These results provide further evidence for reversibility concerning hydride migration. One possibility that finds precedent in Cp* complexes of early transition metals involves hydride migration to the metal with formation of a fulvene intermediate^[17]^ (see Scheme 3 a “CH_3_ hydride migration”). Such an intermediate could subsequently be trapped by electrophilic gold, as seen in a related system based on cobaltocene and an acidic iron species.^[33]^ However, the active participation of gold during hydride transfer cannot yet be ruled out.^[34]^ The notion of reversibility is also in agreement with an FLP‐type activation of E−H bonds in the more congested system based on PCyp_2_Ar^Xyl2^, where small amounts of monometallic Lewis base (**1**) and acid (**2 c**) may form in solution, behaving as an unusual thermally induced bimetallic FLP.^[35]^ In fact, treatment of independently prepared [(PCyp_2_Ar^Xyl2^)Au(NH_3_)](NTf_2_) with **1** provide the same reaction outcome, that is, quantitative formation of equimolar amounts of compounds **5** and **6**. Accordingly, when isotopologue **1*‐d***_**15**_ was used instead, compound **5‐*d***_**15**_ was formed with no deuteration at the hydride position, whereas the reaction between **1** and [(PCyp_2_Ar^Xyl2^)Au(ND_3_)](NTf_2_) yielded the corresponding **5‐*d***_**1**_ deuteride. The basic role of **1** can be played by other strong bases such as ^*t*^BuOK, while weaker bases (e.g. NEt_3_) are barely reactive.^[36]^ Overall, this results support the reduced reactivity of O−H bonds on grounds of the limited oxophilicity of gold (i.e. the related aquo adduct of gold does not form in solution).

In summary, we have demonstrated the possibility for reversible C−H activation and hydride migration from the methyl groups of permethylated cyclopentadienyl rings to a late transition metal. This provides an opportunity to investigate late transition metal catalysed transformations in which methyl groups of the commonly‐employed C_5_Me_5_ ligand act as proton shuttles. The combination of organometallic fragments of opposing Lewis nature was crucial for achieving this reversible migration. It also allowed for the isolation of exotic structures, such as compounds **4** in which the electrophilic gold fragment is anchored to the activated cyclopentadienyl ligand. The intermolecular reactivity of these pairs towards X−H bonds (X=N, O) provides further mechanistic support.

## Conflict of interest

The authors declare no conflict of interest.

## Supporting information

As a service to our authors and readers, this journal provides supporting information supplied by the authors. Such materials are peer reviewed and may be re‐organized for online delivery, but are not copy‐edited or typeset. Technical support issues arising from supporting information (other than missing files) should be addressed to the authors.

Supplementary
