# Supplementary material for: Reversible Hydride Migration from C5Me5 to RhI Revealed by a Cooperative Bimetallic Approach
Source: Angew Chem Int Ed Engl. 2020 Sep 9;59(47):20863–7. doi: 10.1002/anie.202008442 (PMC7754342; doi:10.1002/anie.202008442)
Supplement: Supplementary file 1 — Supplementary [file ANIE-59-20863-s001.pdf]

Angewandte Chemie
Eine Zeitschrift der Gesellschaft Deutscher Chemiker

Supporting Information

Reversible Hydride Migration from C_5Me_5 to Rh^I Revealed by a Cooperative Bimetallic Approach

*Macarena G. Alférez, Juan J. Moreno, Nereida Hidalgo, and Jesús Campos**

anie_202008442_sm_miscellaneous_information.pdf

Supporting Information

Table of Contents

1. Experimental Procedures S2

2. Isotopic labelling of compound **1** S8

3. X-Ray structural characterization of new compounds. S10

4. UV-vis spectra of compounds **1**, **3a**, **4c** and **5** S11

5. Computational details S12

6. NMR spectra of new compounds S13

7. References S24

1. Experimental Procedures

General considerations. All preparations and manipulations were carried out using standard Schlenk and glove-box techniques, under argon or high purity nitrogen atmosphere, respectively. All solvents were dried, stored over 4 Å molecular sieves, and degassed prior to use. Toluene (C_7H_8) and n-pentane (C_5H_{12}) were distilled under nitrogen over sodium. Benzene-d_6 and toluene-d_8 were dried over molecular sieves (4 Å). THF-d_8 was destilled under nitrogen over sodium/benzophenone. Compounds [Au(THT)Cl][1], **1**[2], **2a**[3], **2c**[4] and **5**[2] were prepared according to previously reported procedures. Other chemicals were commercially available and used as received. Solution NMR spectra were recorded on Bruker AMX-300, DRX-400 and DRX-500 spectrometers. Spectra were referenced to external SiMe4 (δ: 0 ppm) using the residual proton solvent peaks as internal standards (^{1}H NMR experiments), or the characteristic resonances of the solvent nuclei (^{13}C{^{1}H} NMR experiments), while ^{31}P was referenced to H_3PO_4. Spectral assignments were made by routine one- and two-dimensional NMR experiments where appropriate. For elemental analyses a LECO TruSpec CHN elementary analyzer was utilized. Infrared spectra were recorded on a Bruker Vector 22 spectrometer in Nujol, while UV-vis spectra were collected on a Perkin Elmer Lambda 750 equipped with a peltier Perkin Elmer PTP-6

Synthesis and characterization of new compounds

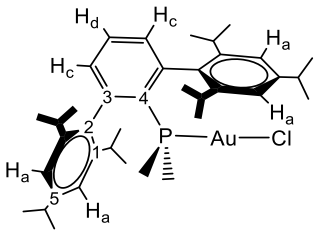

Compound [(*PMe₂Ar*^{Tripp2})AuCl]. A solution of terphenyl phosphine PMe_2Ar^{Tripp2} (510 mg, 0.940 mmol) in toluene (10 mL) was added under nitrogen over a suspension of [Au(THT)Cl] (THT = tetrahydrotiophene) (300 mg, 0.940 mmol) in toluene (5 mL). The initial white suspension was stirred for 8 hours, becoming a solution and then the solvent was removed under vacuum. The resulting white solid was washed with pentane and dried to yield [(PMe_2Ar^{Tripp2})AuCl] as a fine white powder (619 mg, 85%).

^{1}H NMR (400 MHz, $CDCl_3$, 25 ºC) δ: 7.49 (td, 1 H, $^3J_{HH}$ = 7.5 Hz, $^5J_{HP}$ = 2.0, H_d), 7.25 (dd, 2 H, $^2J_{HH}$ = 7.5 Hz, $^4J_{HP}$ = 3.5, H_c), 7.12 (s, 4 H, H_a), 2.99 (hept, 2 H, $^3J_{HH}$ = 6.9 Hz, *p*-(C*H*(CH_3)$_2$), 2.55 (hept, 4 H, $^3J_{HH}$ = 6.8 Hz, *o*-(C*H*(CH_3)$_2$)), 1.35 (m, 24 H, *o, p*-(CH(C*H*$_3$)$_2$)), 1.26 (d, 6 H, $^2J_{HP}$ = 10.3 Hz, PMe_2), 1.06 (d, 12 H, $^3J_{HH}$ = 6.9 Hz, *o*-(CH(C*H*$_3$)$_2$). ^{13}C{^{1}H} NMR (125 MHz, $CDCl_3$, 25 ºC) δ: 150.1 (C_5), 145.9 (C_1), 145.5 (d, $^2J_{CP}$ = 11 Hz, C_3), 135.8 (d, $^3J_{CP}$ = 5 Hz, C_2), 132.7 (d, $^3J_{CP}$ = 8 Hz, CH_c), 129.4 (CH_d), 128.7 (C_4), 121.5 (CH_a), 34.3 (*p*-(*C*H(CH_3)$_2$), 31.3 (*o*-(*C*H(CH_3)$_2$), 25.7 (*o*-(CH(*C*H$_3$)$_2$), 24.3 (*o/p*-(CH(*C*H$_3$)$_2$)), 23.0 (*o/p*-(CH(*C*H$_3$)$_2$)), 17.7 (d, $^1J_{CP}$ = 39 Hz, PMe_2).^{31}P{^{1}H} NMR (202 MHz, CD_2Cl_2, 25 ºC) δ: −5.8.

Anal. Calcd. for $C_{38}H_{55}$AuClP: C, 58.87; H, 7.15. Found: C, 58.5; H, 7.2.

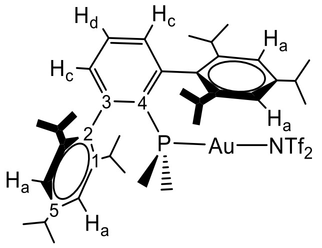

Compound [(PMe_2Ar^{Tripp2})Au(NTf_2)] (2b). A solution of [(PMe_2Ar^{Tripp2})AuCl] (333 mg, 0.430 mmol) in toluene (10 mL) was added over $AgNTf_2$ (167mg, 0.430 mmol) under inert atmosphere. The mixture was stirred at 0ºC for one hour and subsequently filtrated, evaporated and washed with pentane. The residue was dried under vacuum to provide compound **2b** as a temperature-sensitive white solid (145 mg, 40%).

^{1}H NMR (300 MHz, C_6D_6, 25 ºC) δ: 7.42 (m, 1 H, H_d), 7.21 (d, 2 H,$^2J_{HH}$ = 7.5 Hz, H_c), 6.91 (m, 4 H, H_a), 2.95 (hept, 2 H, $^3J_{HH}$ = 7.0 Hz, *p*-(C*H*(CH_3)$_2$)), 2.49 (hept, 4 H, $^3J_{HH}$ = 6.6 Hz, *o*-(C*H*(CH_3)$_2$)), 1.35 (d, 12 H, $^3J_{HH}$ = 7.1 Hz, *p*-(CH(C*H*$_3$)$_2$)), 1.32 (d, 12 H, $^3J_{HH}$ = 6.6Hz, *o*-(CH(C*H*$_3$)$_2$)), 1.06 (d, 6 H, $^2J_{HP}$ = 9.4 Hz, PMe_2), 0.95 (d, 12 H, $^3J_{HH}$ = 6.8 Hz, *o*-(CH(C*H*$_3$)$_2$)). ^{13}C{^{1}H} NMR (100 MHz, C_6D_6, 25 ºC) δ: 150.5 (C_5), 146.3 (C_1), 146.1 (d, $^2J_{CP}$ = 11 Hz, C_3), 135.7 (d, $^3J_{CP}$ = 5 Hz, C_2), 133.1 (d, $^3J_{CP}$ = 8 Hz, CH_c), 130.1 (CH_d),

128.8 (d, $^1J_{CP}$= 76 Hz, C_4), 122.1 (CH_a), 120.0 (q, $^1J_{CF}$ = 320 Hz, CF_3), 34.6 (*p*-(*C*H(CH_3)$_2$)), 31.7 (*o*-(*C*H(CH_3)$_2$)), 25.7 (*o*-(CH(*C*H_3)$_2$)), 24.3 (*p*-(CH(*C*H_3)$_2$)), 22.8 (*o*-(CH(*C*H_3)$_2$)), 17.4 (d, $^1J_{CP}$ = 40 Hz, PMe_2). ^{31}P {^{1}H} NMR (121 MHz, C_6D_6, 25 ºC) δ: −24.6.

Anal. Calcd. for $C_{40}H_{55}AuF_6NO_4PS_2$: C, 47.11; H, 5.44; S, 6.29. Found: C, 47.6; H, 5.3; S, 6.5.

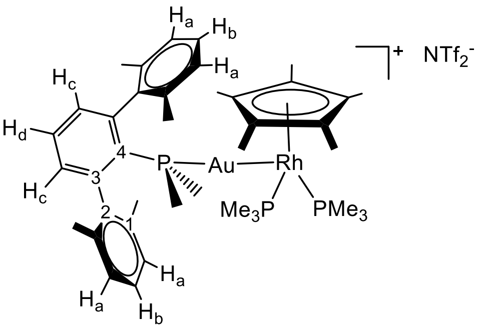

Compound 3a. A solid mixture of compounds **1** (24 mg, 0.061 mmol) and **2a** (50 mg, 0.061 mmol) was dissolved in toluene (5ml) and stirred at room temperature for 30 minutes. Reaction monitoring revealed that formation of **3a** was immediate and proceeded quantitatively by NMR spectroscopy. The solution was concentrated to half of its volume and precipitated with pentane. The residue was then filtered and dried under vacum (49 mg, 66 %). To increase purity, compound **3a** was crystallized by slow diffusion of pentane over a toluene solution to provide a brownish crystalline material.

^{1}H NMR (400 MHz, THF-*d*$_8$, 25 ºC,) δ: 7.60 (td, 1 H, $^3J_{HH}$ = 7.5 Hz, $^3J_{HP}$= 1.9 Hz, H_d), 7.24 (m, 2 H, H_b), 7.13 (m, 6 H, H_c and H_a), 2.27 (s, 12 H, Me_{Xyl}), 1.91 (s, 15 H, C_5Me_5), 1.50 (m, 18 H, PMe_3) 0.96 (d, 6 H, $^3J_{HH}$ = 8.6 Hz, PMe_2). ^{13}C{^{1}H} NMR (100 MHz, THF-*d*$_8$, 25 ºC) δ: 146.1 (d, $^2J_{CP}$ = 9 Hz, C_3), 142.4 (d, $^3J_{CP}$ = 2 Hz, C_2), 137.2 (C_1), 132.2 (d, $^4J_{CP}$ = 3 Hz, CH_d), 131.8 (d, $^3J_{CP}$ = 8 Hz, CH_c), 129.7 (CH_b), 129.2 (d, $^1J_{CP}$ = 77 Hz, C_4), 128.7 (CH_a), 121.2 (q, $^1J_{CF}$ = 323 Hz, CF_3), 101.3 (*C*$_5$Me$_5$), 23.7 (vt, $^1J_{CP}$ = 17 Hz, PMe_3), 22.4 (Me_{Xyl}), 18.1 (d, $^1J_{PC}$ = 33 Hz, PMe_2), 11.8 (C_5*Me*$_5$). ^{31}P{^{1}H} NMR (162 MHz, THF-*d*$_8$, 25 ºC,) δ: 13.9 (td, $^3J_{PP}$ = 12 Hz, $^2J_{PRh}$ = 10 Hz PAu), −3.1 (dd, $^1J_{PRh}$ = 155 Hz, $^3J_{PP}$ = 12 Hz, PMe_3).

Anal. Calcd. For $C_{42}H_{60}AuF_6NO_4P_3RhS_2$: C, 41.56; H, 4.98; N,1.15; S, 5.28. Found: C, 42.04; H, 4.79; N, 1.18; S, 5.42.

UV-vis (Toluene, 1x10^{-4} M): 370 nm.

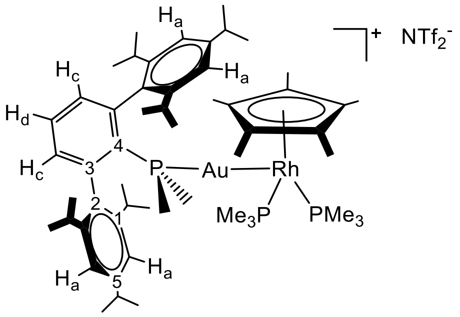

Compound 3b. A solid mixture of compounds **1** (19 mg, 0.049 mmol) and **2b** (50 mg, 0.049 mmol) was dissolved in toluene (5ml) at −60 ºC and stirred for 30 minutes at that temperature. Reaction monitoring revealed that formation of **3b** was immediate and proceeded almost quantitatively (*ca.* 90%) by NMR spectroscopy. The solution was gently warmed up to 25 ºC, concentrated to half of its initial volume and then precipitated with pentane. The residue was then filtered and dried under vacum (30 mg, 44 %). To increase purity, compound **3b** was crystallized by slow diffusion of pentane over a toluene solution to provide a brownish crystalline material.

^{1}H NMR (300 MHz, THF-*d*$_8$, 25 ºC) δ: 7.28 (t, 1 H, $^3J_{HH}$ = 7.6 Hz, H_d), 7.09 to 6.99 (m, 6 H, H_c and H_a), 3.03 (m, 2 H, *p*-(C*H*(CH_3)$_2$)), 2.85 (m, 4 H, *o*-(C*H*(CH_3)$_2$)), 1.63 (s, 15 H, C_5Me_5), 1.53 (d, 12 H, $^3J_{HH}$ = 7.5 Hz, *p*-(CH(C*H*$_3$)$_2$)), 1.44 (d, 12 H, $^3J_{HH}$ = 7.1 Hz, *o*-(CH(C*H*$_3$)$_2$)), 1.38 (t, 18 H, PMe_3), 1.30 (d, 6 H, $^3J_{HH}$ = 8.9 Hz, PMe_2), 1.11 (d, 12 H, $^3J_{HH}$ = 7.2 Hz, *o/p*-(CH(C*H*$_3$)$_2$)). ^{13}C{^{1}H} NMR (100 MHz, C_6D_6, 25 ºC) δ: 150.0 (C_5), 146.8 (C_1), 144.9 (d, $^2J_{CP}$ = 11 Hz, C_3), 137.2 (d, $^3J_{CP}$ = 4 Hz, C_2), 133.2 (d, $^3J_{CP}$ = 7 Hz, CH_c),

131.5 (CH_d), 128.8 (d, $^1J_{CP}$ = 83 Hz, C_4), 120.9 (CH_a), 120.7 (q, $^1J_{CF}$ = 327 Hz, CF_3), 96.0 (C_5Me_5), 34.6 (*p*-($CH(CH_3)_2$))), 31.7 (*o*-($CH(CH_3)_2$)), 25.8 (*o*-($CH(CH_3)_2$)), 24.1 (*p*-($CH(CH_3)_2$)), 23.2 (vt,$^1J_{CP}$ = 17 Hz, PMe_3), 22.7 (*o*-($CH(CH_3)_2$)), 14.3 (d, $^1J_{CP}$ = 33 Hz, PMe_2), 11.7 (C_5Me_5). $^{31}P\{^1H\}$ NMR (121 MHz, THF-d_8, 25 ºC,) δ: 15.2 (dt, $^3J_{PP}$ = 14 Hz, $^2J_{PRh}$ = 12 Hz PMe_2), −5.1 (dd, $^1J_{PRh}$ = 155 Hz, $^3J_{PP}$ = 14 Hz, PMe_3).

Anal. Calcd. for $C_{56}H_{88}AuF_6O_5P_3RhS_2$: C, 47.70; H, 6.29; N, 0.99; S, 4.55. Found C, 47.71; H, 6.20; N, 1.31; S, 4.58.

Compound 4b. Compound **4b** cannot be isolated in pure form as it represents the minor isomer during the reaction of **1** and **2b** (maximun conversion of around 30%, *c.f.* major isomer **3b**: *ca.* 70%) and exhibits a pronouced reactivity and thermal unstability. Nevertheless, multinuclear NMR analysis permitted identification of its ^1H and $^{31}P\{^1H\}$ NMR resonances in isomeric mixtures of **3b**:**4b**.

1H NMR (400 MHz, THF-d^8, 25 ºC,) δ: 7.26 to 6.90 (aromatic-CH), 2.73 (m, 2 H, *p*-($CH(CH_3)_2$)), 2.55 (m, 4 H, *o*-($CH(CH_3)_2$)), 1.60 (s, 12 H, C_5Me_5), 1.48 (d, 12 H, $^3J_{HH}$ = 7.5 Hz, *p*-($CH(CH_3)_2$)), 1.39 (d, 12 H, $^3J_{HH}$ = 7.1 Hz, *o*-($CH(CH_3)_2$)), (d, 6 H, $^3J_{HH}$ = 8.9 Hz, PMe_2), 1.30 (t, 18 H, PMe_3), 1.07 (d, 12 H, $^3J_{HH}$ = 7.2 Hz, *o*-($CH(CH_3)_2$)), −13.5 (dt, 1 H, $^2J_{HP}$ = 35.8 Hz, $^1J_{HRh}$ = 24.1 Hz, RhH). $^{31}P\{^1H\}$ NMR (162 MHz, THF-d_8, 25 ºC,) δ: 12.7 (dt, $^3J_{PP}$ = 12 Hz , PMe_2), −2.2 (dd, $^1J_{PRh}$ = 140 Hz, $^3J_{PP}$ = 12 Hz, PMe_3).

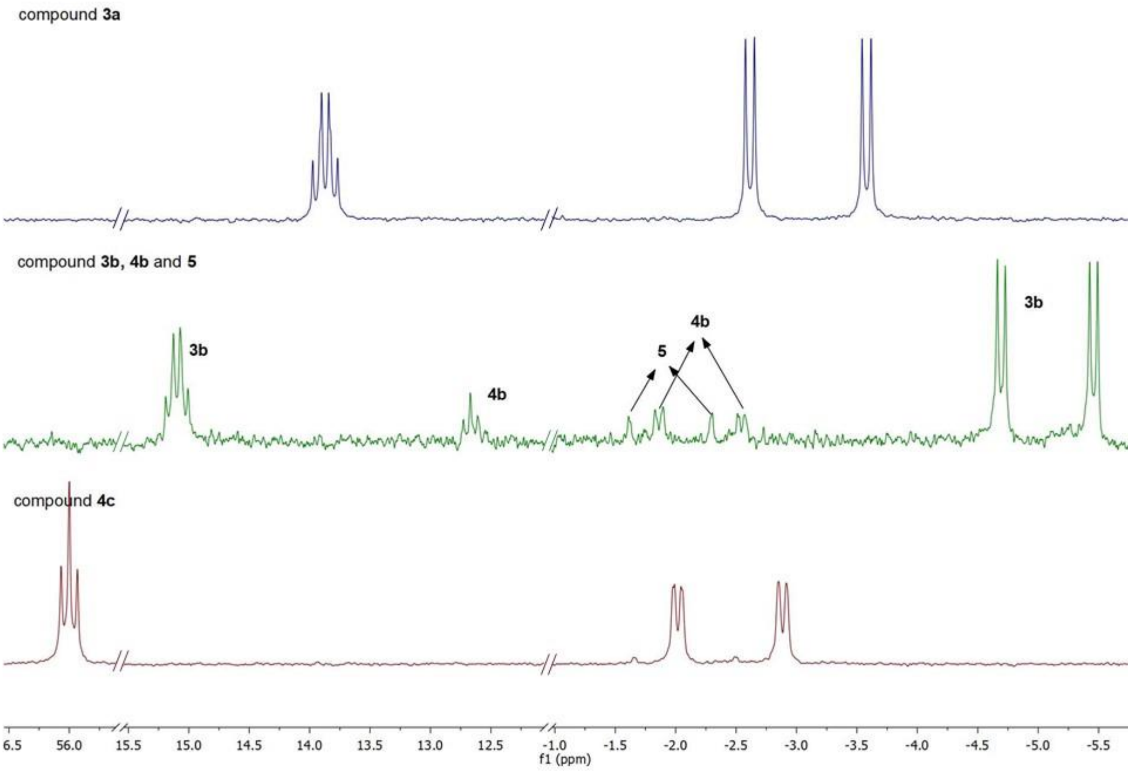

Figure S1. Selected regions of $^{31}P\{^1H\}$ NMR stack spectra of compounds **3** and **4**.

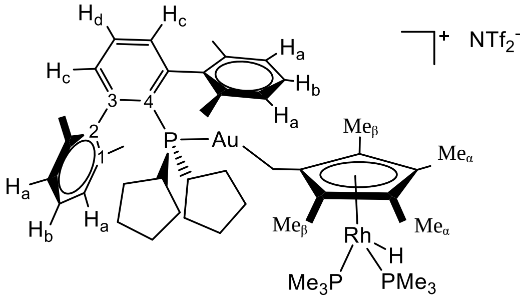

Compound 4c. A solid mixture of compounds **1** (21 mg, 0.054 mmol) and **2c** (50 mg, 0.054 mmol) was dissolved in toluene (5ml) and stirred for 30 minutes under argon atmosphere at 25 ºC. Reaction monitoring revealed that formation of **4c** was immediate and proceeded almost quantitatively (*ca.* 95%) by NMR spectroscopy. The solution was concentrated to *ca.* half of its original volume and the bimetallic product was precipitated with pentane. The residue was filtered dried under vacum (108 mg, 81 %). For better purity, compound **4c** was crystallized by slow diffusion of pentane into a toluene solution to provide a brownish crystalline material.

^{1}H NMR (400 MHz, THF-d_8, 25 ºC) δ: 7.59 (t, 1 H, $^3J_{HH}$ = 7.6 Hz, H_d), 7.21 (m, 2 H, H_b), 7.11 (m, 6 H, H_c and H_a), 2.24 (m, 2 H, Cyp(CH)), 2.06 (s, 12 H, Me_{Xyl}), 1.84 (s, 6 H, Me_α), 1.73 (s, 6 H, Me_β) 1.55 (m, 8 H, Cyp(CH_2)), 1.48 (m, 18 H, PMe_3), 1.39 (m, 8 H, Cyp(CH_2)), 1.05 (d, 2 H, $^2J_{HP}$ = 9.6 Hz, CH_2-Au), −13.3 (dt, 1 H, $^2J_{HP}$ = 35.8 Hz, $^1J_{HRh}$ = 24.5 Hz, RhH). ^{13}C{^{1}H} NMR (100 MHz, THF-d_8, 25 ºC) δ: 149.5 (d, $^2J_{CP}$ = 10 Hz, C_3), 143.2 (d, $^3J_{CP}$ = 5 Hz, C_2) 137.3 (C_1), 133.0 (d, $^3J_{CP}$ = 6 Hz, CH_c), 132.1 (d, $^4J_{CP}$ = 4 Hz, CH_d), 129.6 (d, $^1J_{CP}$ = 46 Hz, C_4), 128.6 (CH_a), 128.3 (CH_b), 121.2 (q, $^1J_{CF}$ = 322 Hz, CF_3), 102.7 (*C*CH_2), 100.7 (*C*Me_α), 91.6 (*C*Me_β), 38.6 (d, $^1J_{CP}$ = 28 Hz, Cyp(CH)), 35.3 (d, $^3J_{CP}$ = 9 Hz, Cyp(CH_2)), 32.8 (d, $^3J_{CP}$ = 10 Hz, Cyp(CH_2)), 26.4 (d, $^2J_{CP}$ = 10 Hz, Cyp(CH_2)), 21.9 (Me_{Xyl}), 21.7 (d, $^2J_{CP}$ = 10 Hz, Cyp(CH_2)), 20.3 (t, $^2J_{CP}$ = 17 Hz, PMe_3), 11.5 and 11.3 (Me_α and Me_β), 1.3 (d, $^2J_{CP}$ = 3 Hz, C*C*H_2). ^{31}P {^{1}H} NMR (162 MHz, THF-d_8, 25 ºC) δ: 56.0 (t, $^5J_{PP}$ = 10 Hz, PMe_2), −2.2 (dd, $^1J_{PRh}$ = 140 Hz, $^5J_{PP}$ = 10, PMe_3).

Anal. Calcd. For $C_{51}H_{76}AuF_6NO_4P_3RhS_2$: C, 45.78; H, 5.73; S, 4.79; N, 1.04. Found: C, 45.84; H, 5.22; S, 5.27; N, 1.15.

UV-vis (Toluene, 1x10^{-4} M): 305 nm.

X-H (X = H, C, O, N) bond activation studies using compounds 1 and 2. All the reactions were carried out using the same experimental procedure. A solution of compound **1** and **2** (**2c:** 8.0 mg, **2b**: 9.0 mg; 0.009 mmol) in either C_6D_6 or THF-d_8 (0.6 mL) in a J. Young NMR tube. The solution was shaken and then the corresponding liquid (H_2O, MeOH, NH_3 solution in dry THF; 1 equivalent) or gas (NH_3, 0.5 bar) reagent was added and the reaction progress monitored by ^{1}H and ^{31}P{^{1}H} NMR spectroscopy. Assignment of the resulting gold and rhodium products was made on the basis of the independently synthesized gold compounds (see below) and the known spectroscopic signatures of compound **5**.[1] The following superimposed ^{31}P{^{1}H} NMR spectra due to the isomeric mixture **3b:4b** evinces that only compounds of type **4** are reactive towards polar E—H bonds, while metal adducts **3** remains unaltered.

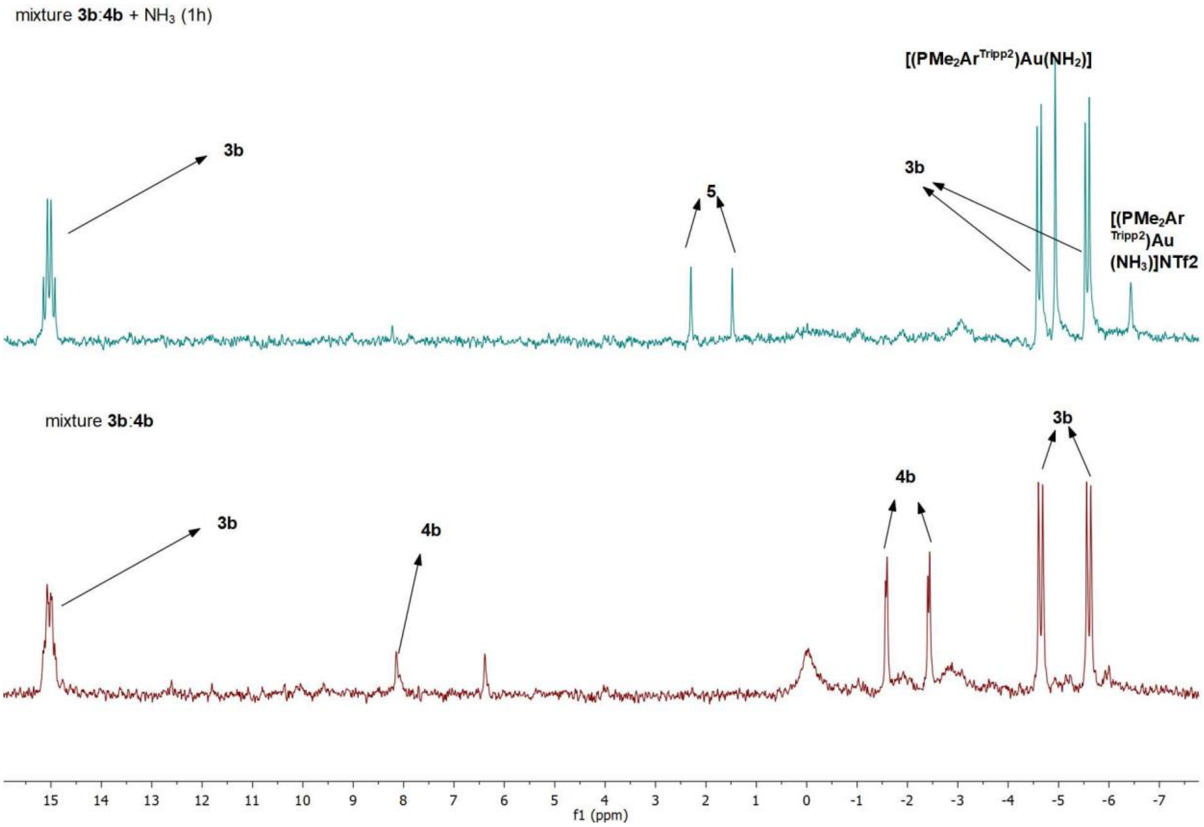

Figure S2. ^{31}P{^{1}H} stack NMR spectra of the isomeric mixture **3b**:**4b** before and after exposure to NH_3 (0.5 bar).

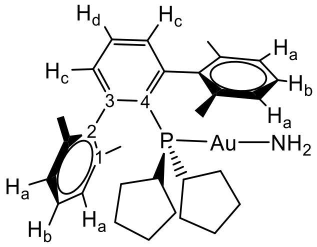

Compound 6 [(PCyp$_2$Ar^{Xyl2})Au(NH$_2$)]. A solution of **2c** (50 mg, 0.054 mmol) in toluene (5 ml) was treated with one equivalent of LiNH$_2$ (1.2 mg, 0.054 mmol) and the suspension stirred at 25 ºC under argon for 24 hours. The resulting solution was evaporated and extracted with pentane (4 x 5 mL) to yield a mixture of two species in ca 3:1 ratio. While the major compound is attributed to **6** (whose spectroscopic resonances are reported and assigned below), the minor species is ascribed to cation [(PCyp$_2$Ar^{Xyl2})Au(NH$_3$)]$^+$ (also described below). Although we did not separate the two species, we estimate an overall yield for **6** of around 64 %.

^{1}H NMR (500 MHz, C$_6$D$_6$, 25 ºC) δ: 7.44 to 7.21 (m, 7 H, H$_a$, H$_d$, H$_c$), 6.85 (m, 2 H, H$_b$), 2.51 (s, 2 H, NH$_2$), 2.31 (s, 2 H, Cyp(CH)), 2.15 (s, 12 H, Me$_{Xyl}$), 1,92 to 1.77 (m, 8 H, Cyp(CH$_2$)), 1.61 (s, 8 H, Cyp(CH$_2$)). ^{13}C{^{1}H} NMR (100 MHz, C$_6$D$_6$, 25 ºC,) δ: 147.9 (br,C$_3$), 144.4 (br,C$_2$), 137.3 (C$_1$), 132.3 (d, $^3J_{CP}$ = 7 Hz, CH$_c$), 132.1 (d, $^4J_{CP}$ = 4 Hz, CH$_d$),131.6 (CH$_b$), 129.3 (C$_4$), 125.7 (CH$_a$), 38.2 (d, $^1J_{CP}$ = 36 Hz, Cyp(CH)), 35.6 (Cyp(CH$_2$)), 32.8 (Cyp(CH$_2$)), 25.2 (d, $^2J_{CP}$ = 11 Hz, Cyp(CH$_2$)), 21.5 (s, Me$_{Xyl}$). ^{31}P {^{1}H} NMR (202 MHz, C$_6$D$_6$, 25 ºC) δ: 49.8. IR (Nujol): ν_{NH} = 3257, 3311 cm^{-1}.

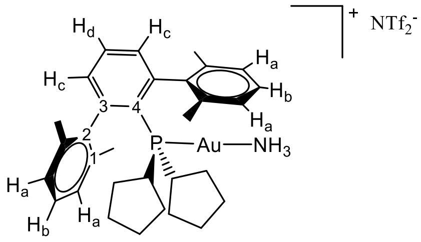

Compound [(PCyp$_2$Ar^{Xyl2})Au(NH$_3$)]NTf$_2$ A solution of **2c** (50 mg, 0.054 mmol) in toluene (5 ml) was placed under dry ammonia atmosphere (0.5 bar) in a pressure vessel and the solution stirred for 24 hours. Then it was evaporated and washed up with pentane (3 x 5 mL) to yield compound [(PCyp$_2$Ar^{Xyl2})Au(NH$_3$)]NTf$_2$ as a white powder (30 mg, 59 %). Isotopologue [(PCyp$_2$Ar^{Xyl2})Au(ND$_3$)]NTf$_2$ could be prepared in similar yield by adding ND$_3$/D$_2$O (25% wt, 1 mL) to a solution of **2c** (50 mg, 0.054 mmol) in THF (5 mL) and stirring for 3 hours at 25 ºC. Solvent evaporation was followed by several washings with pentane to provide the targeted ammonia isotopologue as a white powder.

^{1}H NMR (400 MHz, C$_6$D$_6$, 25 ºC) δ: 7.26 to 7.03 (m, 7 H, H$_a$, H$_d$, H$_c$), 6.63 (m, 2 H, H$_b$), 2.55 (s, 3 H, NH$_3$), 2.04 (s, 2 H, Cyp(CH)), 1.96 (s, 12 H, Me$_{Xyl}$), 1.83 (s, 4 H, Cyp(CH$_2$)), 1.70 (s, 2 H, Cyp(CH$_2$)), 1.59 (s, 2 H, Cyp(CH$_2$)), 1.40 (s, 8 H, Cyp(CH$_2$)). ^{13}C{^{1}H} NMR (100 MHz, C$_6$D$_6$, 25 ºC) δ: 148.1 (br,C$_3$), 144.5 (br,C$_2$), 137.3 (C$_1$), 132.2 (d, $^3J_{CP}$ = 7 Hz, CH$_c$), 131.6 (CH$_d$), 128.6 (CH$_b$), 120.9 (q, $^1J_{CF}$ = 320 Hz, CF$_3$), 38.1 (d, $^2J_{CP}$ = 36 Hz, Cyp(CH)), 35.6 (d, $^3J_{CP}$ = 6 Hz, Cyp(CH$_2$)), 32.8 (d, $^3J_{CP}$ = 6 Hz, Cyp(CH$_2$)), 25.2 (d, $^2J_{CP}$ = 116 Hz, Cyp(CH$_2$)) 25.1 (d, $^2J_{CP}$ = 13 Hz, Cyp(CH$_2$)), 21.3 (s, Me$_{Xyl}$). Signals due to C$_4$ and CH$_a$ could not be precisely assigned. ^{31}P{^{1}H} NMR (162 MHz, C$_6$D$_6$, 25 ºC) δ: 50.2. IR (Nujol): ν_{NH} = 3186, 3271, 3378 cm^{-1}.

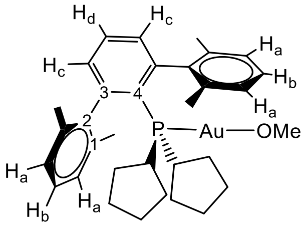

Compound 7, **[(PCyp$_2$Ar^{Xyl2})Au(OMe)].** A solution of **2c** (50 mg, 0.054 mmol) in toluene (5 ml) was treated with one equivalent of CH$_3$ONa (2.9 mg, 0.054 mmol) and the solution stirred for 24 hour. Then it was evaporated and extracted with toluene (4 x 5 mL). The filtrated was dried under vacuum and the residue washed with pentane to yield compound **7** as a white powder (20 mg, 56 %).

^{1}H NMR (400 MHz, C_6D_6, 25 ºC) δ: 7.32 (s, 2 H, H_c), 7.16 to 6.96 (m, 5 H, H_a, H_d), 6.62 (s, 2 H, H_b), 2.11 (s, 2 H, Cyp(CH)), 1.95 (s, 12 H, Me_{Xyl}), 1.70 to 1.14 (m, 16 H, Cyp(CH_2)). ^{13}C{^{1}H} NMR (100 MHz, C_6D_6, 25 ºC) δ: 148.1 (br,C_3), 142.3 (br,C_2), 137.3 (C_1), 132.3 (d, $^2J_{CP}$ = 7 Hz, C_c), 131.2 (CH_d), 128,9 (d, $^1J_{CP}$ =72 Hz, C_4), 127.4 (CH_a), 125.7 (CH_b), 38.3 (d, $^1J_{CP}$ = 37 Hz, Cyp(CH)), 35.7 (d, $^2J_{CP}$ = 6 Hz, Cyp(CH_2)), 33.0 ((OMe)), 25.1 (d, $^3J_{CP}$ = 12 Hz, Cyp(CH_2)), 21.5 (Me_{Xyl}). ^{31}P{^{1}H} NMR (162 MHz, C_6D_6, 25 ºC) δ: 47.7.

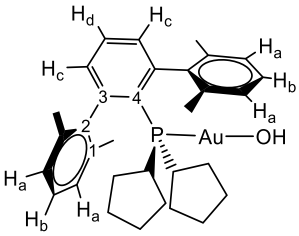

Compound 8 [(PCyp$_2$Ar^{Xyl2})Au(OH)].A solution of compound [(PCyp$_2$Ar^{Xyl2})AuCl] (50 mg, 0.072mmol) in benzene (5 ml) was treated with one equivalent of KOH (4.0 mg, 0.072 mmol) and the suspension stirred at room temperature for 24 hours. The resulting solution was then evaporated and extracted with pentane (4 x 5 mL). The filtrate was dried under reduced pressure to provide compound **8** as a white powder (28 mg, 57 %).

^{1}H NMR (400 MHz, C_6D_6, 25 ºC) δ: 7.20 (q, 2 H, $^3J_{HH}$ = 7.1 Hz, H_b), 7.07 (d, 4 H, $^2J_{HH}$ = 7.5 Hz, H_a), 6.92 (td, 1 H, $^3J_{HH}$ = 7.4 Hz, $^5J_{HP}$ = 1.6 Hz, H_d), 6.55 (dd, 2 H, $^2J_{HH}$ = 7.6 Hz, $^2J_{HP}$ = 3.2 Hz, H_c), 2.02 to 1.85 (m, 2 H, Cyp(CH)), 1.90 (s, 12 H, Me_{Xyl}), 1.65 to 1.15 (m, 16 H, Cyp(CH_2)). ^{13}C{^{1}H} NMR (100 MHz, C_6D_6, 25 ºC,) δ: 148.1 (br, C_3), 142.3 (br, C_2), 136.4 (C_1), 131.7 (d, $^3J_{CP}$ = 7 Hz, CH_c), 130.4 (CH_d), 128.4 (CH_b), 127.5 (CH_a), 38.1 (d, $^2J_{CP}$ = 36 Hz, Cyp(CH)), 34.8 (d, $^3J_{CP}$ = 6 Hz, Cyp(CH_2)), 32.2 (d, $^3J_{CP}$ = 6 Hz, Cyp(CH_2)), 24.9 (d, $^2J_{CP}$ = 16 Hz, Cyp(CH_2)) 24.8 (d, $^2J_{CP}$ = 13 Hz, Cyp(CH_2)), 21.3 (s, Me_{Xyl}). Signals due to C_4 could not be precisely assigned. ^{31}P{^{1}H} NMR (162 MHz, C_6D_6, 25 ºC) δ: 47.7. IR (Nujol): ν_{OH} = 3503 cm^{-1}.

2. Isotopic labelling of compound 1

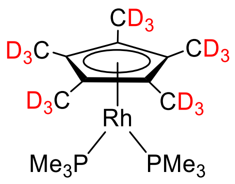

1-*d*$_{15}$

Deuteration of the cyclopentadienyl ligand in compound 1. Isotopologue **1-*d*$_{15}$** was best prepared by heating a CD_3OD/Toluene (1:1, 4 mL overall) solution of **1** (100 mg, 0,257 mmol) at 90 ºC for 30 hours under argon atmosphere. Evaporation under vacum and washing with pentane led to the corresponding labelled compound as a brown powder (52 mg, 50 %). A first-order kinetic profile (Figure S4) was obtained by monitoring the reaction by ^{1}H NMR spectroscopy (Figure S3) under slighlty different conditions (**1**: 6.5 mg, 0.017 mmol; CD_3OD 0.6 mL; 90ºC).

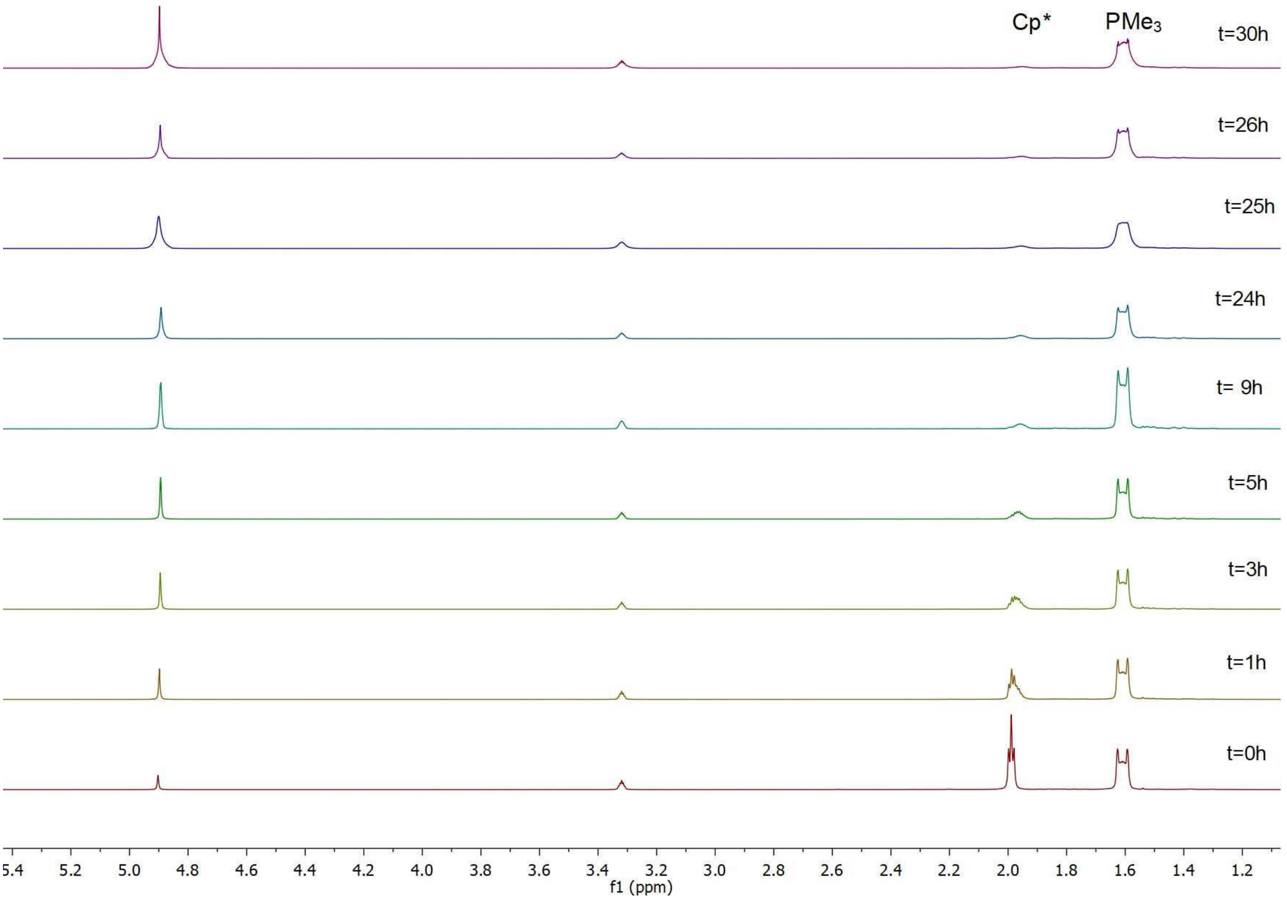

Figure S3. ^{1}H NMR monitoring of the deuteration the cyclopentadienyl ligand in compound **1**

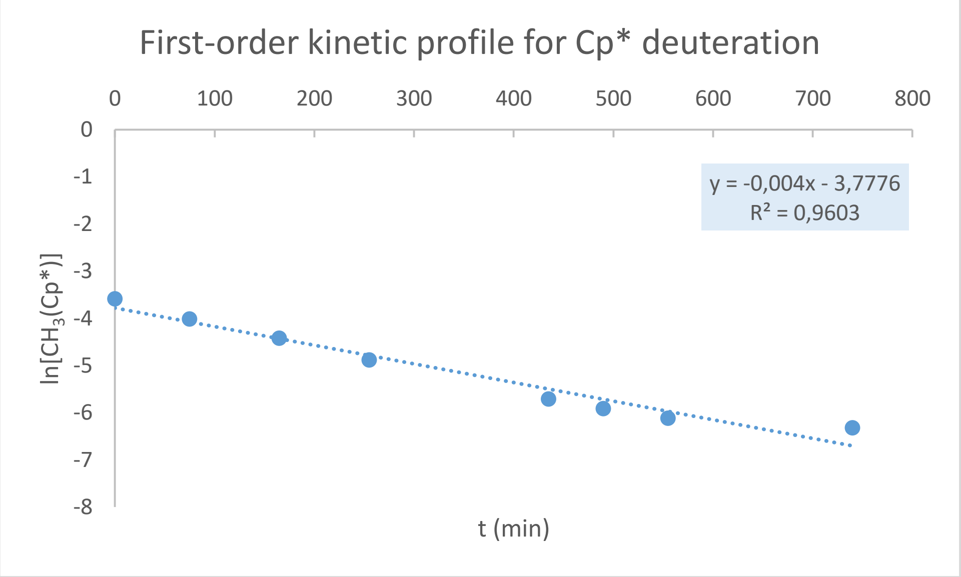

Figure S4. First order kinetic representation for the deuteration of the cyclopentadienyl ligand in **1**, accounting for a k_1 of $6.66 \cdot 10^{-5}$ s^{-1}.

3. X-Ray structural characterization of new compounds

Crystallographic details. Crystals of compounds **3b** and **4c** were grown by slow diffusion of pentane into their benzene or THF solutions, respectively. Low-temperature diffraction data were collected on a Bruker APEX-II CCD diffractometer using monochromatic radiation λ(Mo $K_{\alpha 1}$) = 0.71073 Å at the Instituto de Investigaciones Químicas de Sevilla. Data collections were processed with APEX-W2D-NT (Bruker, 2004), cell refinement and data reduction with SAINT-Plus (Bruker, 2004)[5] and the absorption was corrected by multiscan method applied by SADABS.[6] The structures were solved with SHELXT and was refined against F^2 on all data by full-matrix least squares with SHELXL.[7] All non-hydrogen atoms were refined anisotropically. Hydrogen atoms were included in the model at geometrically calculated positions and refined using a riding model. The isotropic displacement parameters of all hydrogen atoms were fixed to 1.2 times the U value of the atoms to which they are linked (1.5 times for methyl groups). The hydride ligand directly bound to rhodium in structure **4c** was located at the difference electron density map and its Rh—H bond distance restrained to typical values. The two structures contain solvent molecules in the unit cell (benzene and pentane in **3b** and THF in **4c**) with variable degrees of disorder to which several restraints were applied.

A summary of the fundamental crystal and refinement data are given in Table S1. Atomic coordinates, anisotropic displacement parameters and bond lengths and angles can be found in the cif files, which have been deposited in the Cambridge Crystallographic Data Centre with no. 2008917 and 2008918. These data can be obtained free of charge from The Cambridge Crystallographic Data Centre via www.ccdc.cam.ac.uk/data_request/cif.

Table S1. Crystal data and structure refinement for compounds **3b** and **4c**

	Compound 3b	Compound 4c
**formula**	C73 H112 Au F6 N O4 P3 Rh S2	C58 H88 Au F6 N O6 P3 Rh S2
**fw**	1638.54	1466.20
**cryst.size, mm**	0.19 x 0.15 x 0.14	0.18 x 0.14 x 0.08
**crystal system**	triclinic	triclinic
**space group**	*P-1*	*P-1*
***a*, Å**	14.604(3)	12.9888(18)
***b*, Å**	15.299(3)	14.845(2)
***c*, Å**	17.639(3)	18.299(3)
***α*, deg**	87.555(8)	87.190(7)
***β*, deg**	89.702(8)	75.660(7)
***γ*, deg**	87.674(9)	70.153(7)
**V, Å^3**	3934.0(12)	3213.0(8)
***T*, K**	193(2)	193(2)
**Z**	2	2
**ρ_{calc}, g cm^{-3}**	1.383	1.516
**μ, mm^{-1} (MoKα)**	2.25	2.75
***F*(000)**	1688	1492
**absorption corrections**	multi-scan,0.60-0.75	multi-scan, 0.61-0.75
**θ range, deg**	2.7 – 29.3	2.3 – 24.7
**no. of rflns meads**	118646	39254
**R_{int}**	0.029	0.037
**no. of rflns unique**	18016	11597
**no. of params / restraints**	847 / 70	720 / 117
**R_1 ($I > 2\sigma(I)$) ^{a}**	0.029	0.054
**R_1 (all data)**	0.036	0.078
**wR_2 ($I > 2\sigma(I)$)**	0.083	0.131
**wR_2 (all data)**	0.090	0.145
**Diff.Fourier.peaks min/max, eÅ^{-3}**	-1.14 / 1.82	-1.69 / 2.88
**CCDC number**	2008918	2008917

4. UV-vis spectra of compounds 1, 3a, 4c and 5

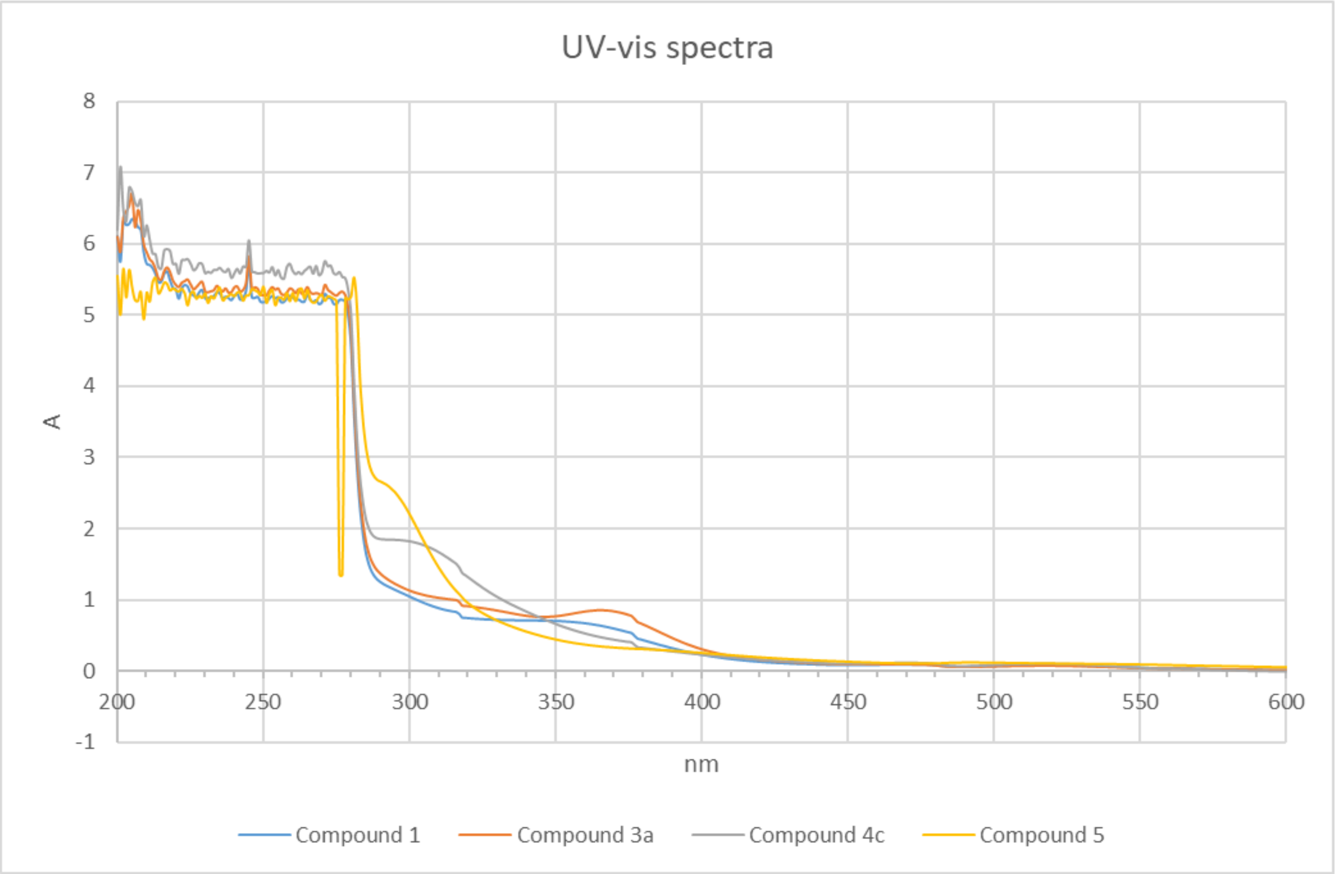

Figure S5. Stacked representation of UV-vis spectra of compounds **1**, **3a**, **4c** and **5**.

5. Computational details

General details.
DFT calculations were performed with the Gaussian software package.[8] Geometry optimization of minima was carried out without symmetry restrictions using the hybrid functional PBE0;[9] dispersion effects were accounted for by using Grimme's D3 parameter set with Becke−Johnson (BJ) damping.[10] The 6-31g(d,p) basis set[11] was used for non-metal atoms, Au and Rh atoms were described with the SDD basis and associated electron core potential (ECP).[12] Bulk solvent effects (dichloromethane) were included during optimization with the SMD continuum model.[13] Frequency calculations were carried out to ensure minima presented zero imaginary vibrational frequencies. Calculated geometries were in excellent agreement with crystallographic data. AIM charges, also known as Bader charges, were computed according to QTAIM[14] by means of the Multiwfn program,[15] using high quality grid (grid spacing = 0.06 Bohr) to generate basins and locate attractors. Integration of real space functions in AIM basins was carried out with mixed type of grids, using exact refinement of basin boundary to improve accuracy.

AIM Charge analysis.
We found of interest to interrogate the charge distribution on the metals of relevant examples of the reported bimetallic complexes as well as on their monometallic precursors. To do so we have examined compounds **3a** and **4c**, as well as the monometallic species **1**, **5** and [$(PM_2Ar^{Xyl2})Au(NTf_2)$]. The results of AIM charge analysis are collected in Table S2. The formally Au(I) centre in [$(PMe_2Ar^{Xyl2})Au(NTf_2)$] presents an AIM charge of 0.19. For [$Cp^*Rh(PMe_3)_2$] (**1**) the formally Rh(I) metal center bears an AIM charge of -0.12, a value that rises to 0.21 upon protonation to produce a formally Rh(III) species in **5**. In this compound, the Rh-bound hydride possesses an AIM charge of -0.15. Very similar values (0.19 for Rh, -0.17 for the hydride) are obtained for complex **4c**, in which the AIM charge of the Au atom becomes more negative (-0.04). This can be ascribed to the stronger donating ability of the [$C_5Me_4CH_2$]$^-$ anion relative to the triflimide. Interestingly, in the bimetallic Lewis adduct **3a**, the Rh centre presents an intermediate AIM charge of 0.08, whereas the Au atom, despite being bound to a neutral fragment, displays a more negative charge of -0.17. Overall, these results along with the UV-vis spectra collected in Figure S5, highlight the difficulties of unambiguously assigning formal oxidation states in bimetallic species of type **3**.

Table S2. AIM charges of relevant mono and bimetallic species at gold, rhodium and hydride centres.

	**Au**	**Rh**	Rh—***H***
**[$(PMe_2^{Xyl2})Au(NTf_2)$]**	0.19		
**1**		-0.12	
**5**		0.21	-0.15
**4c**	-0.04	0.19	-0.17
**3a**	-0.17	0.08	

6. NMR spectra of new compounds

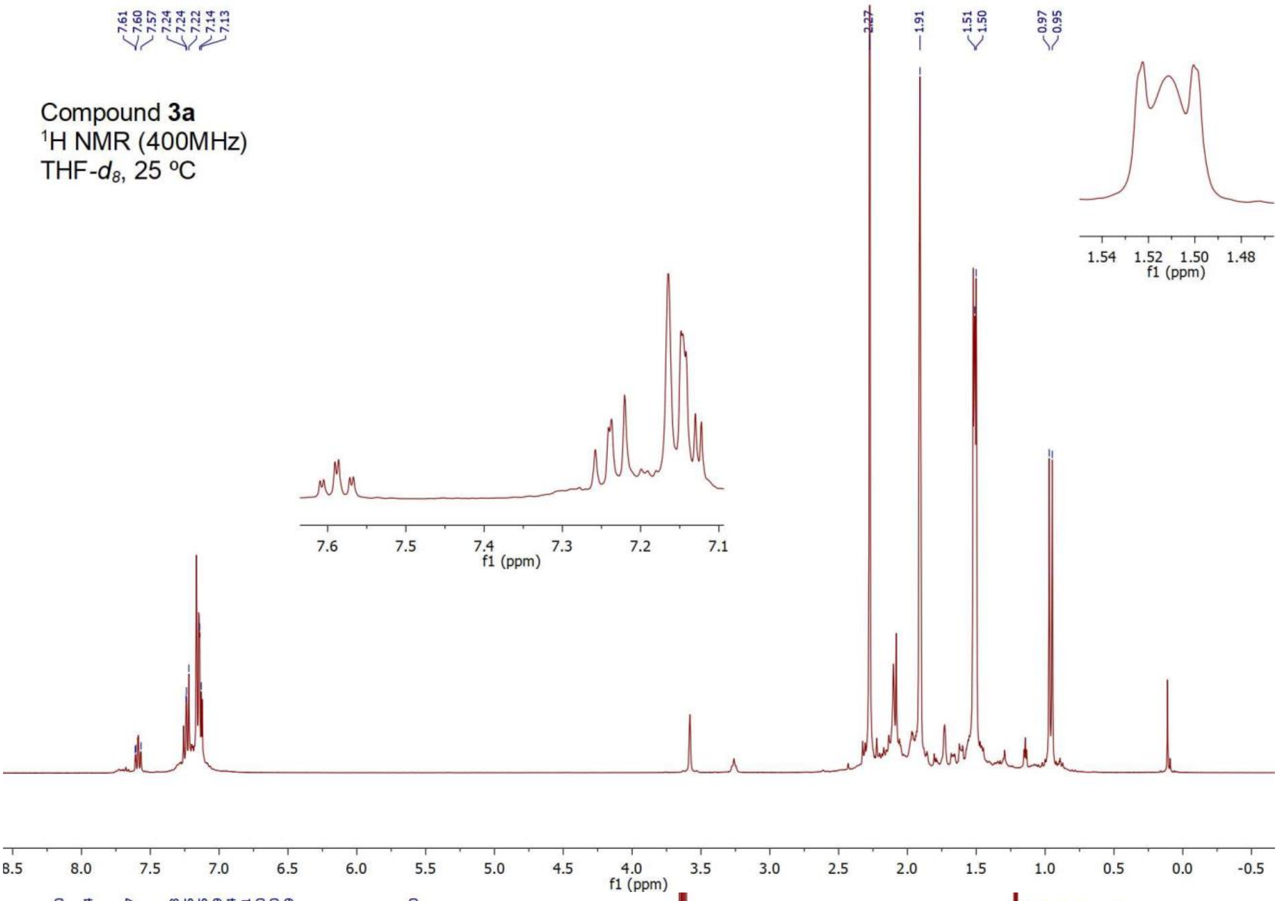

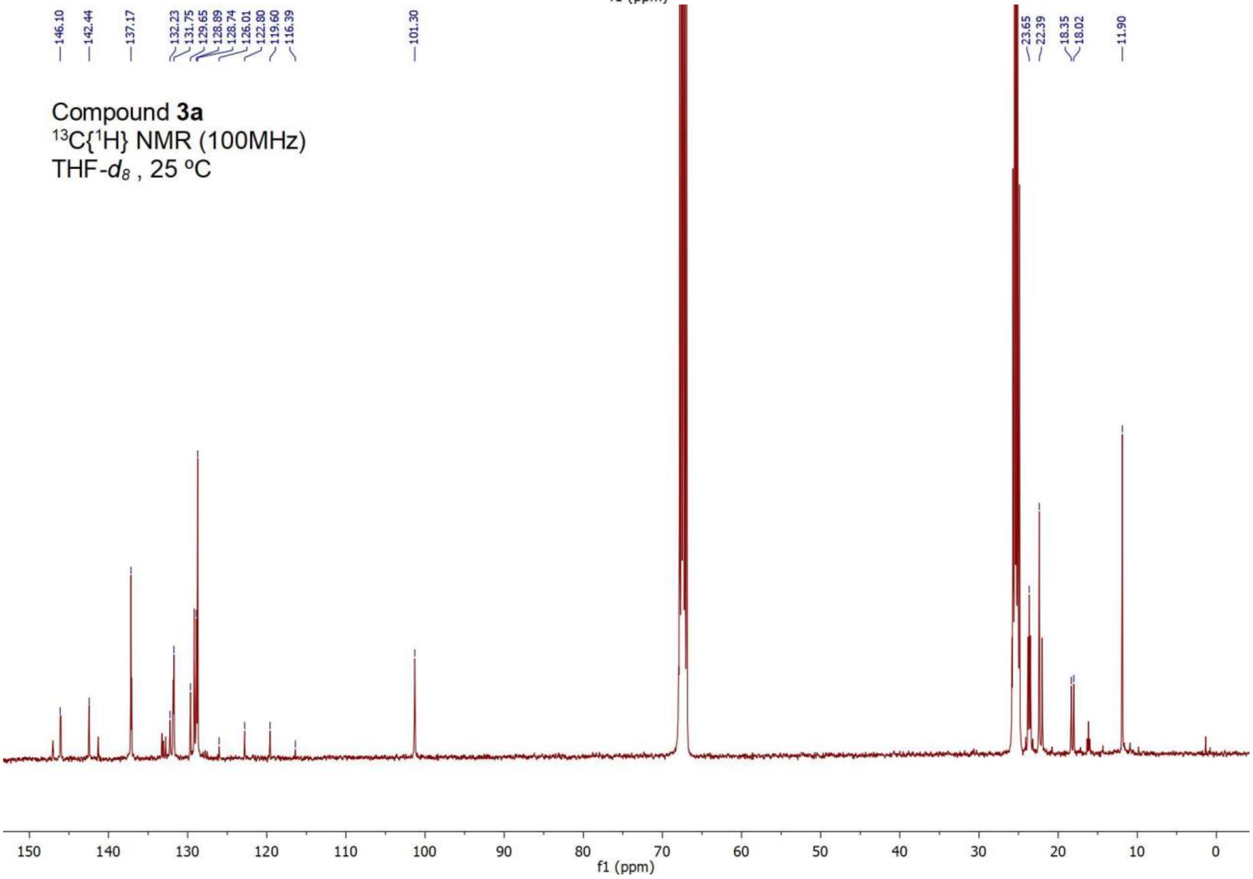

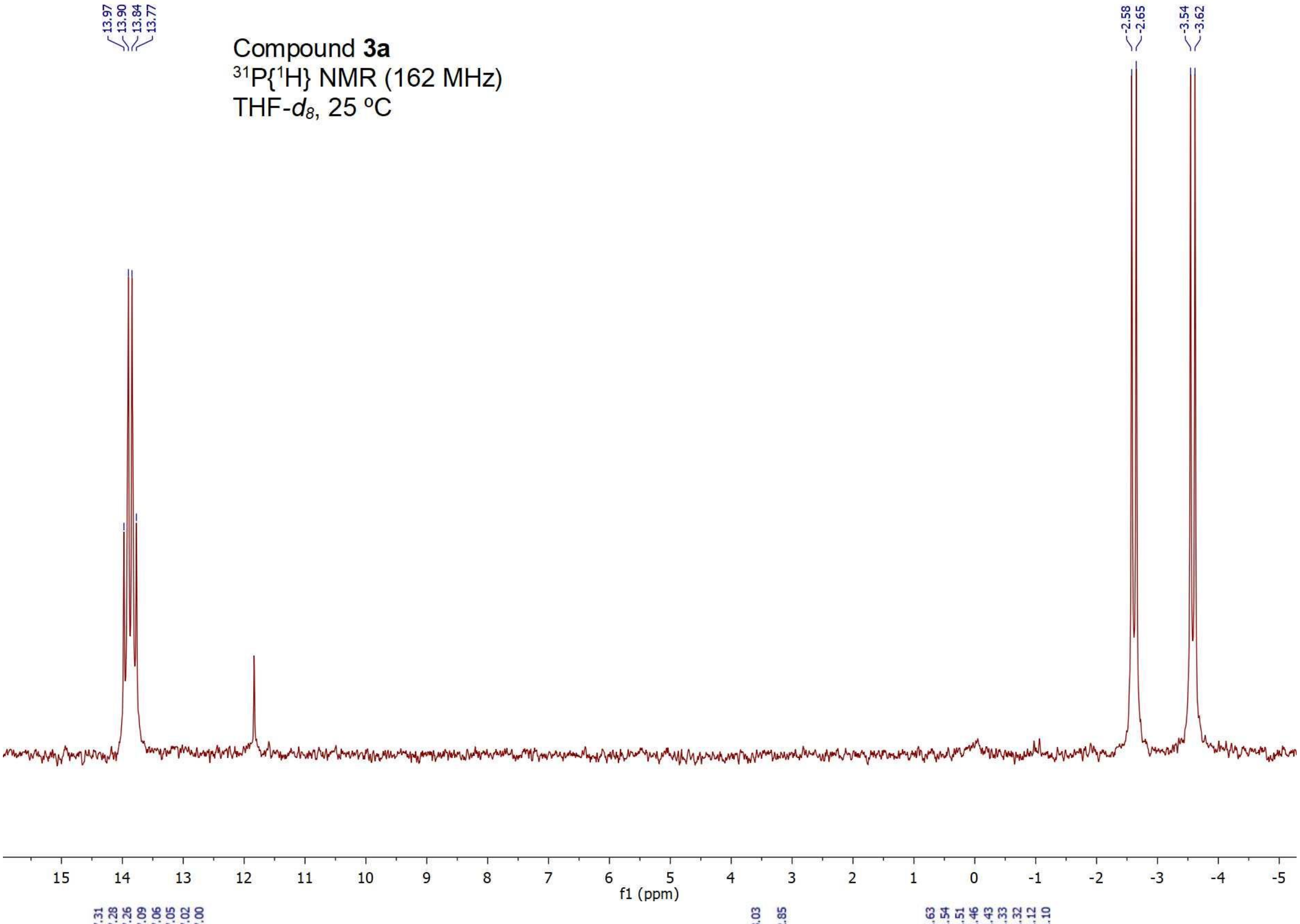
13.97
13.90
13.84
13.77
-2.58
-2.65
-3.54
-3.62
Compound 3a
31P{1H} NMR (162 MHz)
THF-d8, 25 ºC
15
14
13
12
11
10
9
8
7
6
5
4
3
2
1
0
-1
-2
-3
-4
-5
f1 (ppm)

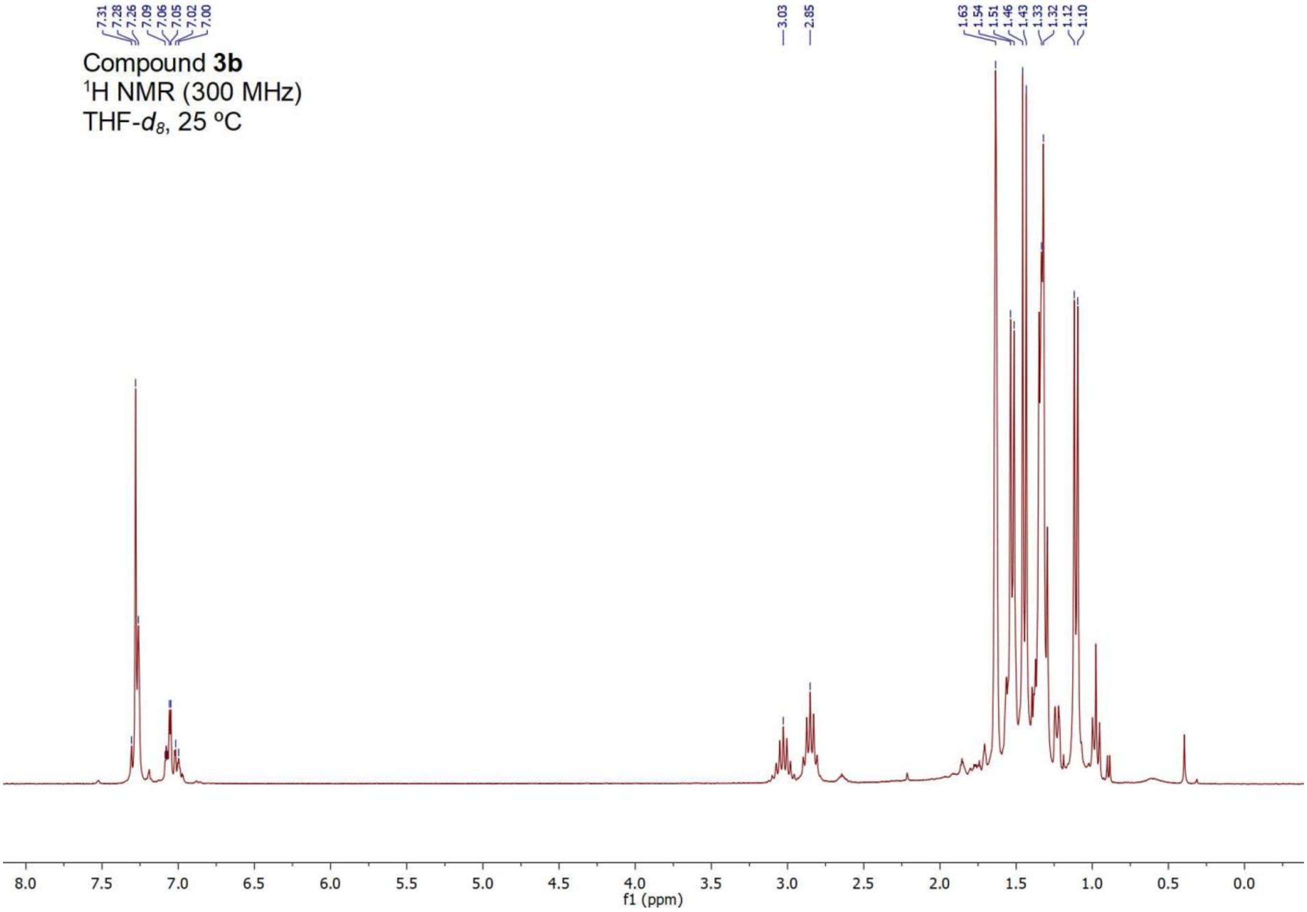
7.31
7.28
7.26
7.09
7.06
7.05
7.02
7.00
3.03
2.85
1.63
1.54
1.51
1.46
1.43
1.33
1.32
1.12
1.10
Compound 3b
1H NMR (300 MHz)
THF-d8, 25 ºC
8.0
7.5
7.0
6.5
6.0
5.5
5.0
4.5
4.0
3.5
3.0
2.5
2.0
1.5
1.0
0.5
0.0
f1 (ppm)

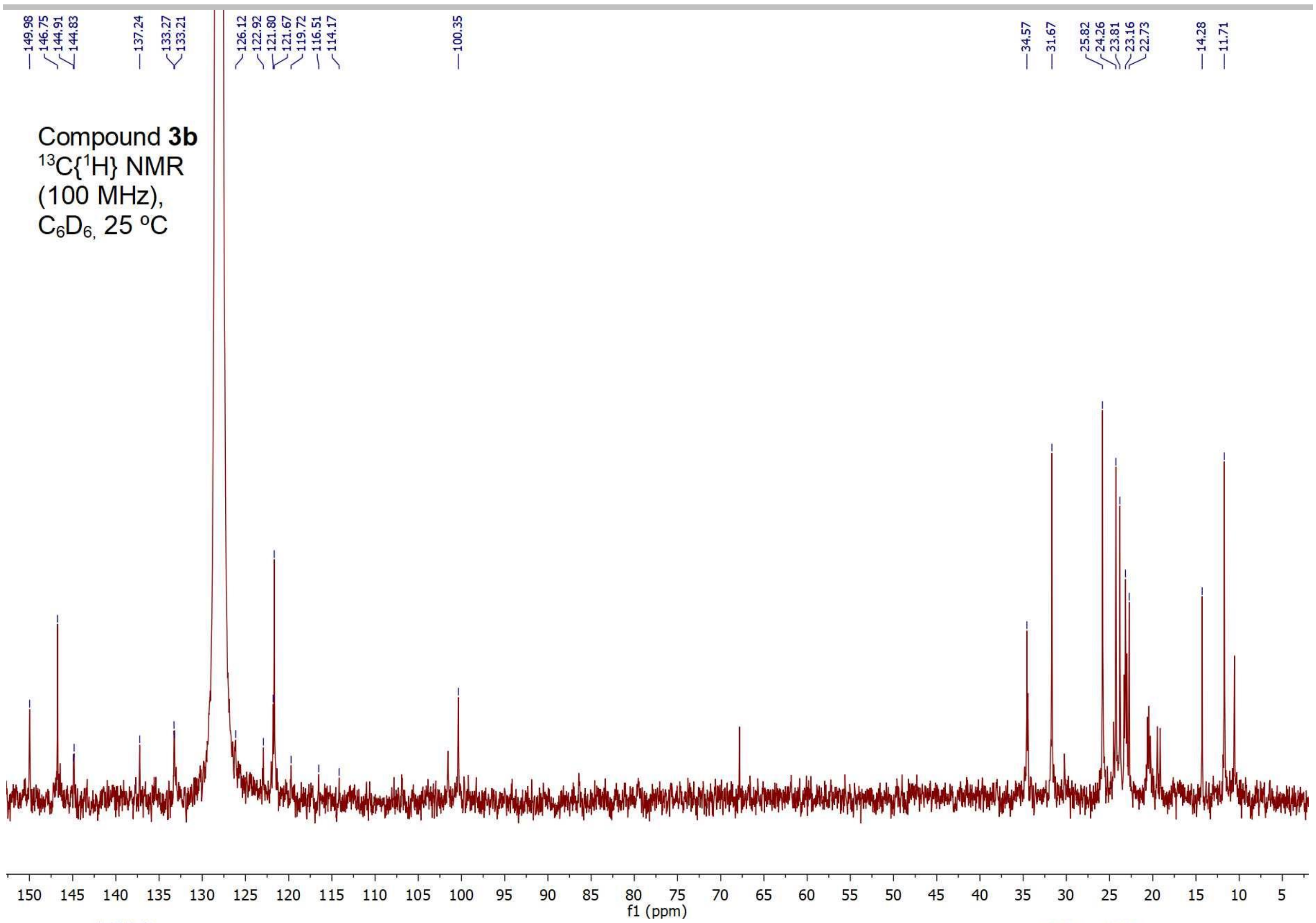
149.98
146.75
144.91
144.83
137.24
133.27
133.21
126.12
122.92
121.80
121.67
119.72
116.51
114.17
100.35
34.57
31.67
25.82
24.26
23.81
23.16
22.73
14.28
11.71
Compound **3b**
$^{13}C\{^1H\}$ NMR
(100 MHz),
C_6D_6, 25 °C
f1 (ppm)

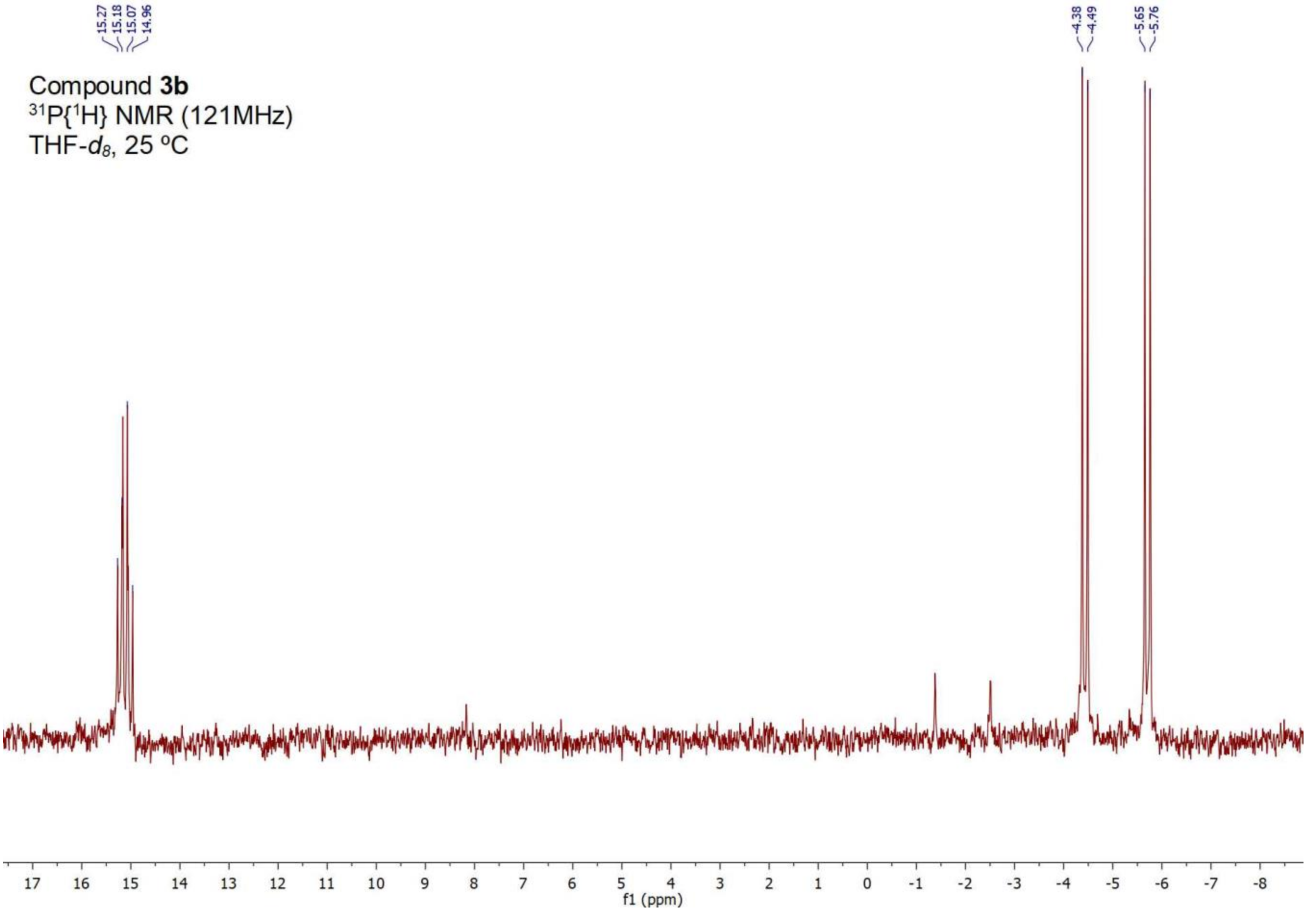
15.27
15.18
15.07
14.96
-4.38
-4.49
-5.65
-5.76
Compound **3b**
$^{31}P\{^1H\}$ NMR (121MHz)
THF-d_8, 25 °C
f1 (ppm)

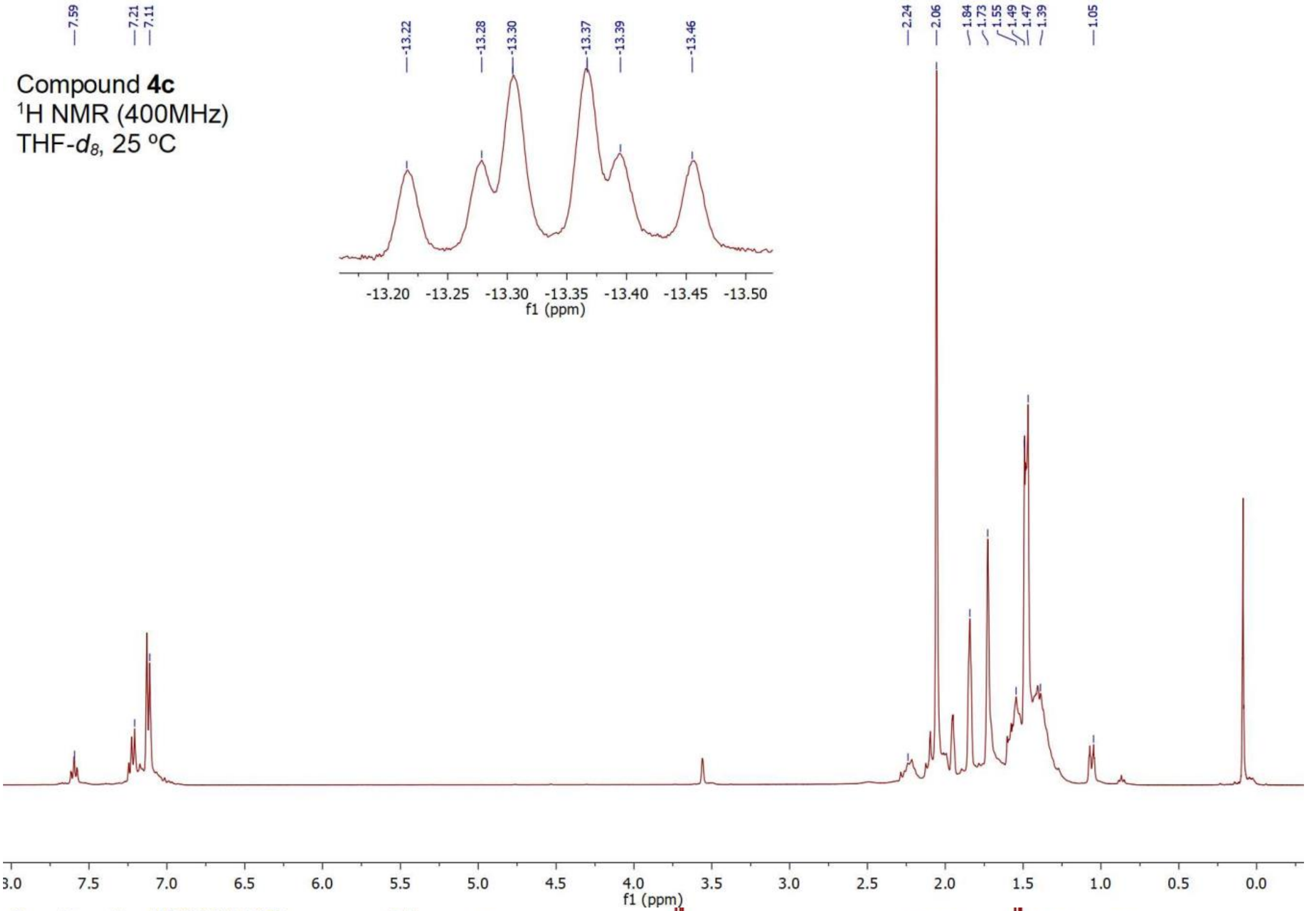
Compound **4c**
^{1}H NMR (400MHz)
THF-d_8, 25 °C
7.59
7.21
7.11
-13.22
-13.28
-13.30
-13.37
-13.39
-13.46
2.24
2.06
1.84
1.73
1.55
1.49
1.47
1.39
1.05
f1 (ppm)

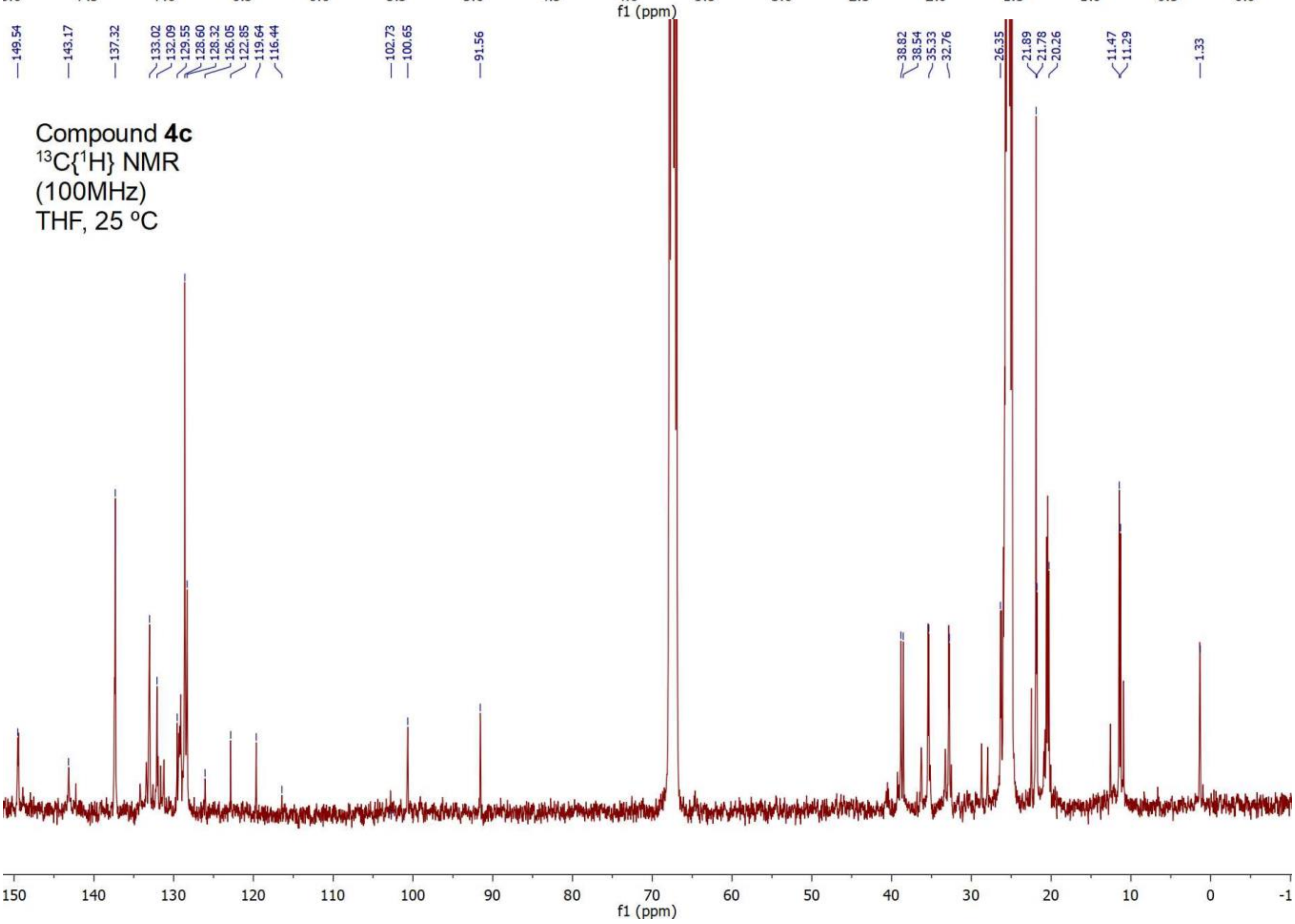
Compound **4c**
^{13}C{^{1}H} NMR
(100MHz)
THF, 25 °C
149.54
143.17
137.32
133.02
132.09
129.55
128.60
128.32
126.05
122.85
119.64
116.44
102.73
100.65
91.56
38.82
38.54
35.33
32.76
26.35
21.89
21.78
20.26
11.47
11.29
1.33
f1 (ppm)

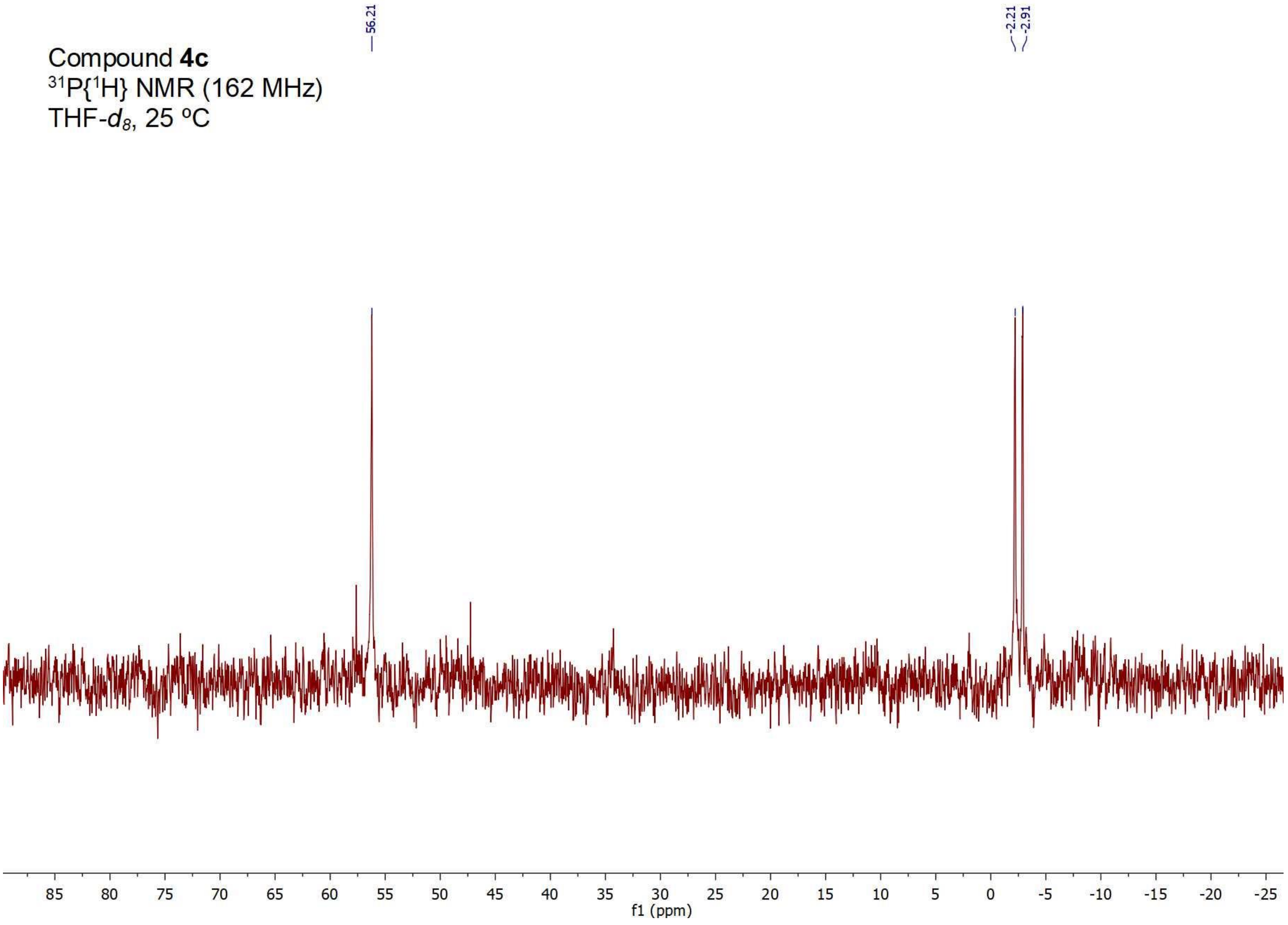
Compound **4c**
$^{31}P\{^{1}H\}$ NMR (162 MHz)
THF-d_8, 25 ºC
56.21
-2.21
-2.91
f1 (ppm)

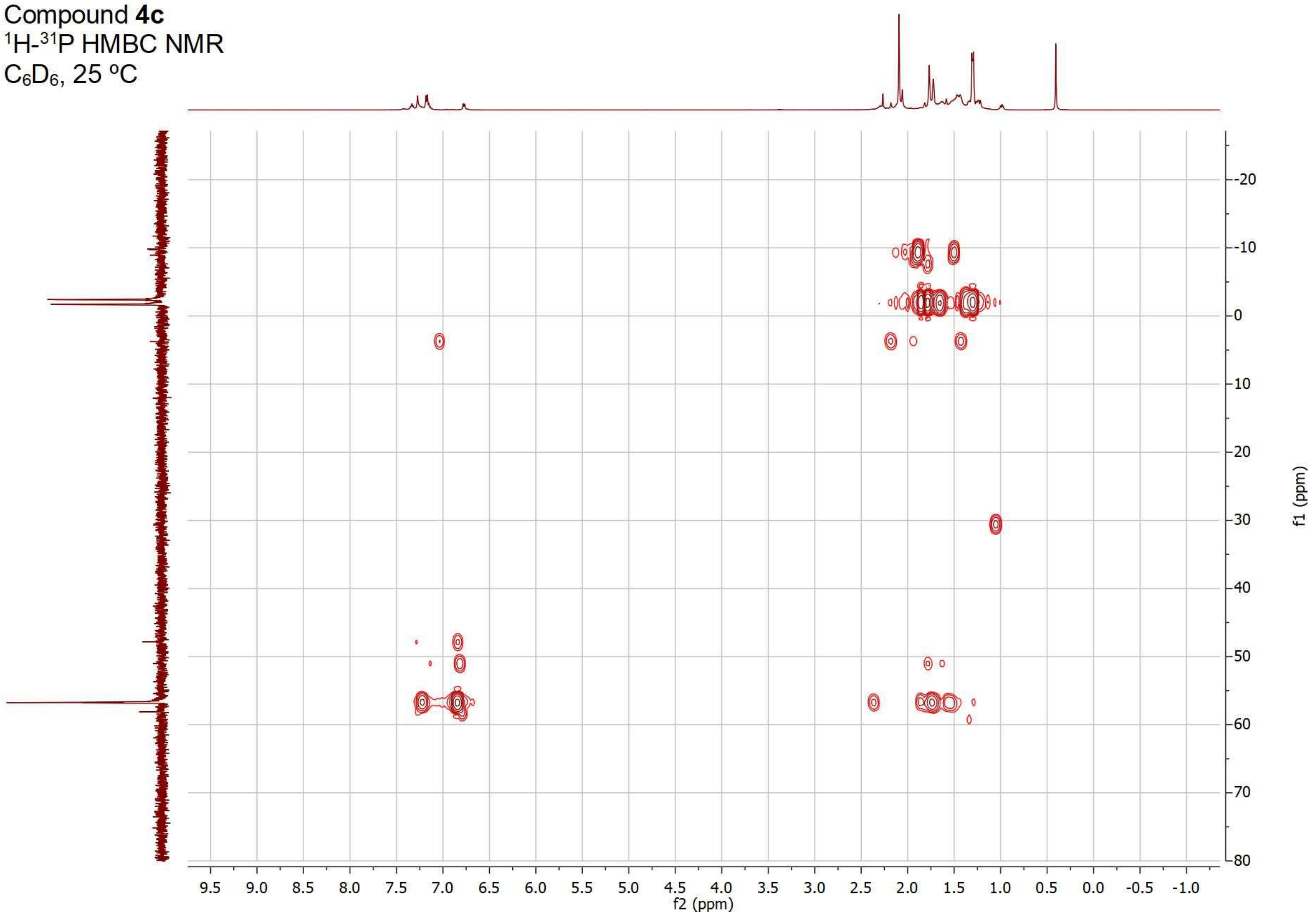
Compound **4c**
^{1}H-^{31}P HMBC NMR
C_6D_6, 25 ºC
f2 (ppm)
f1 (ppm)

Compound **4c**
Dosy NMR
C_6D_6, 25 ºC

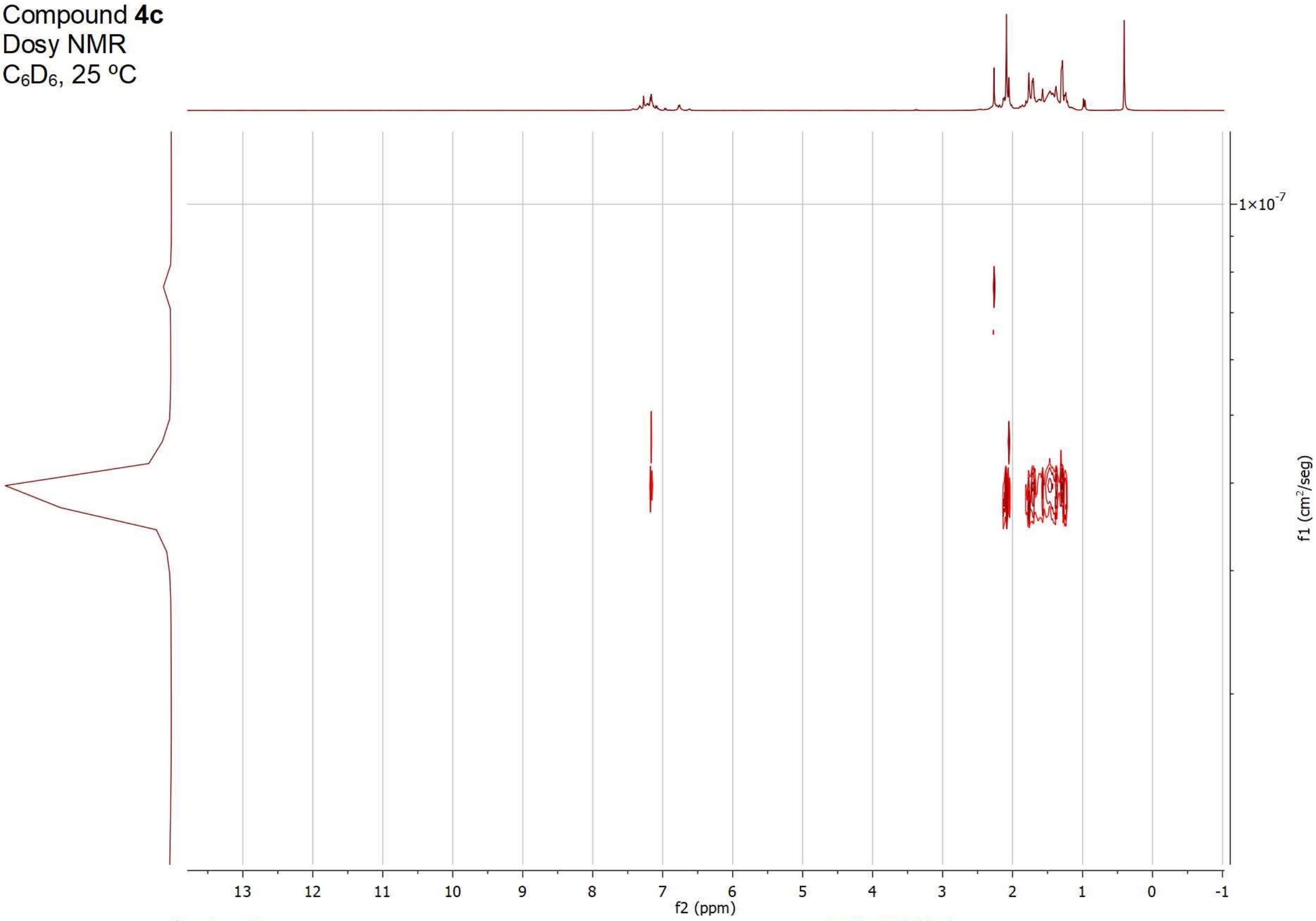

Compound **6**
^{1}H NMR (400 MHz)
C_6D_6,25 ºC

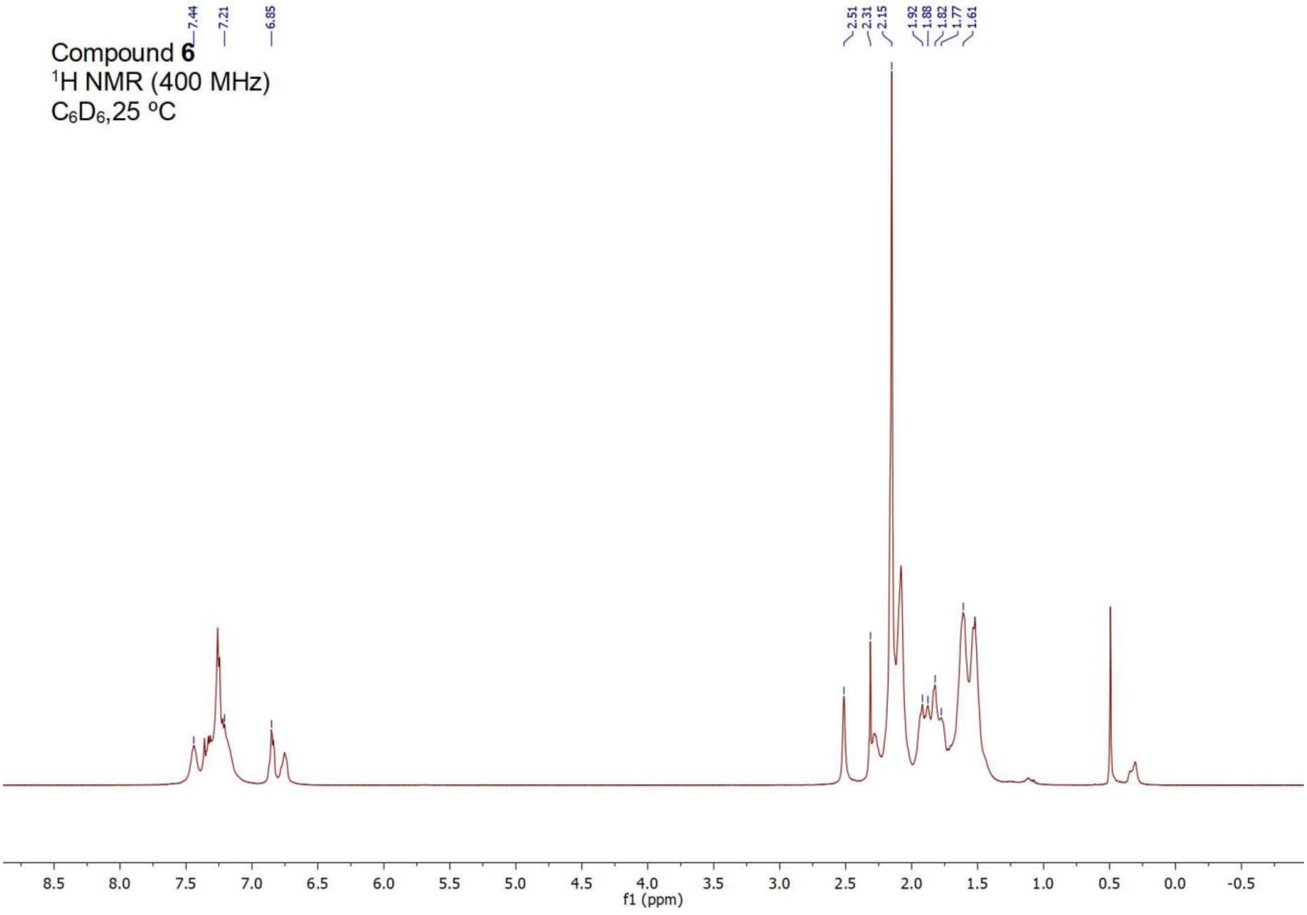

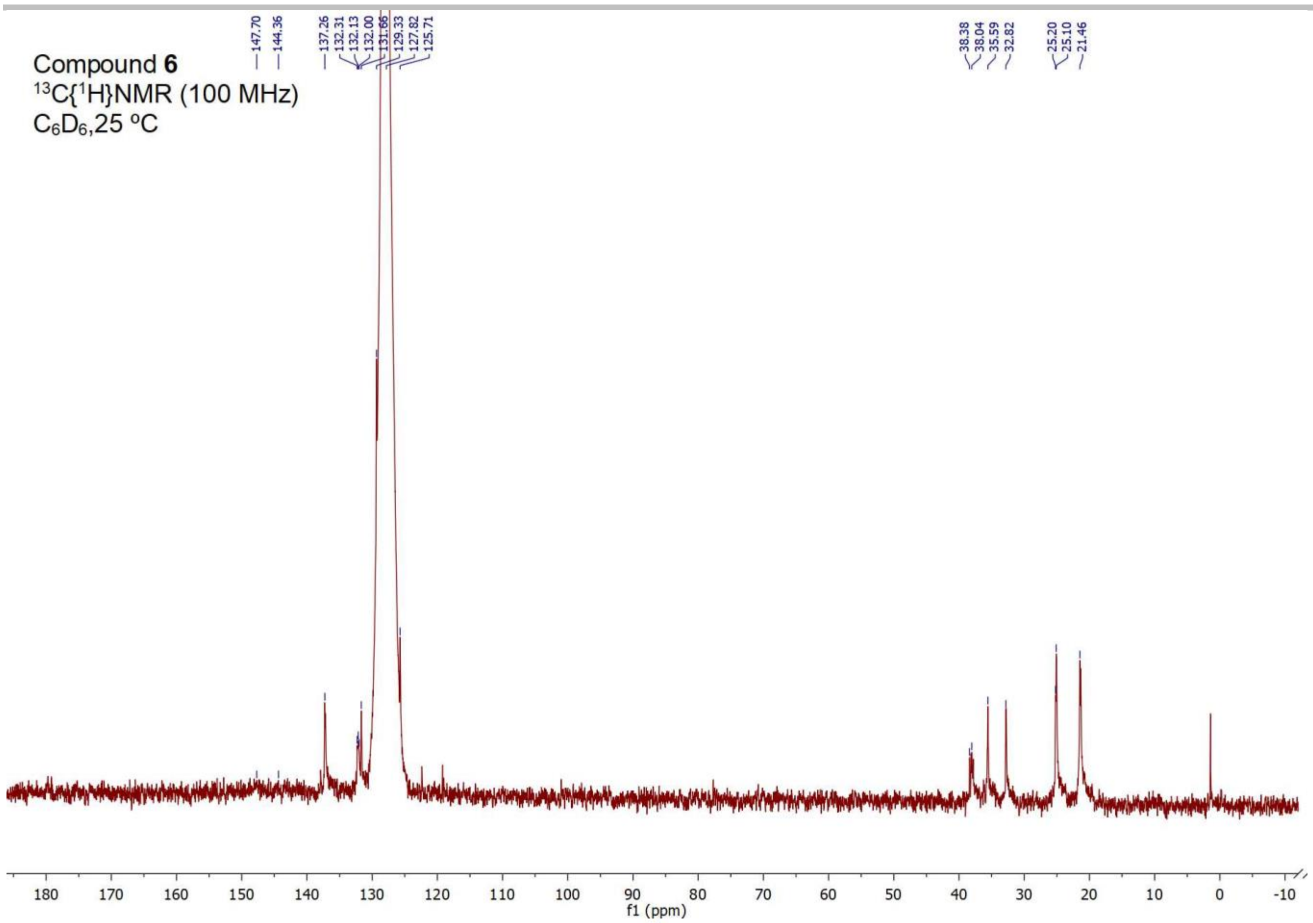
Compound **6**
$^{13}C\{^1H\}$NMR (100 MHz)
C_6D_6,25 ºC
147.70
144.36
137.26
132.31
132.13
132.00
131.66
129.33
127.82
125.71
38.38
38.04
35.59
32.82
25.20
25.10
21.46
180 170 160 150 140 130 120 110 100 90 80 70 60 50 40 30 20 10 0 -10
f1 (ppm)

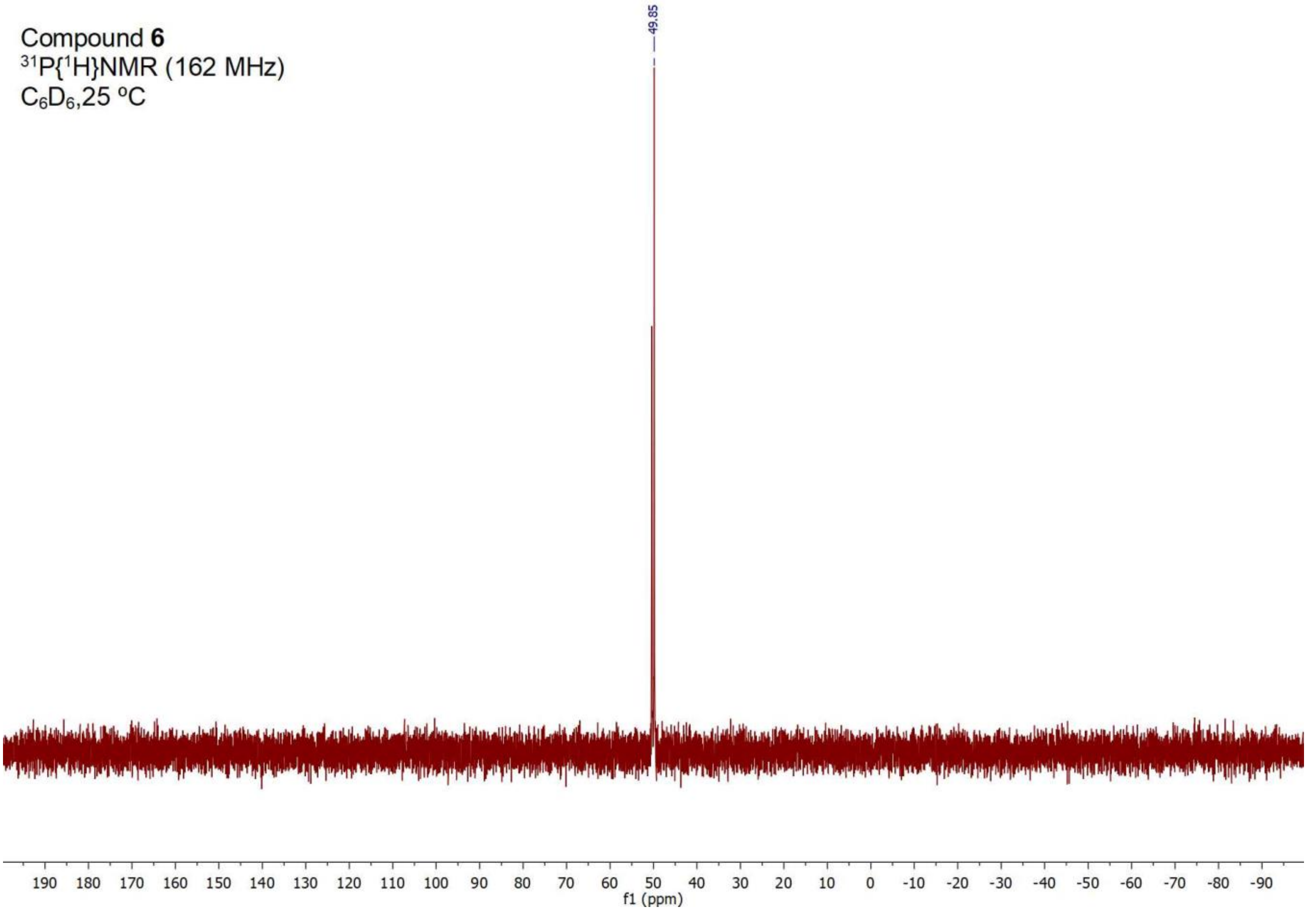
Compound **6**
$^{31}P\{^1H\}$NMR (162 MHz)
C_6D_6,25 ºC
49.85
190 180 170 160 150 140 130 120 110 100 90 80 70 60 50 40 30 20 10 0 -10 -20 -30 -40 -50 -60 -70 -80 -90
f1 (ppm)

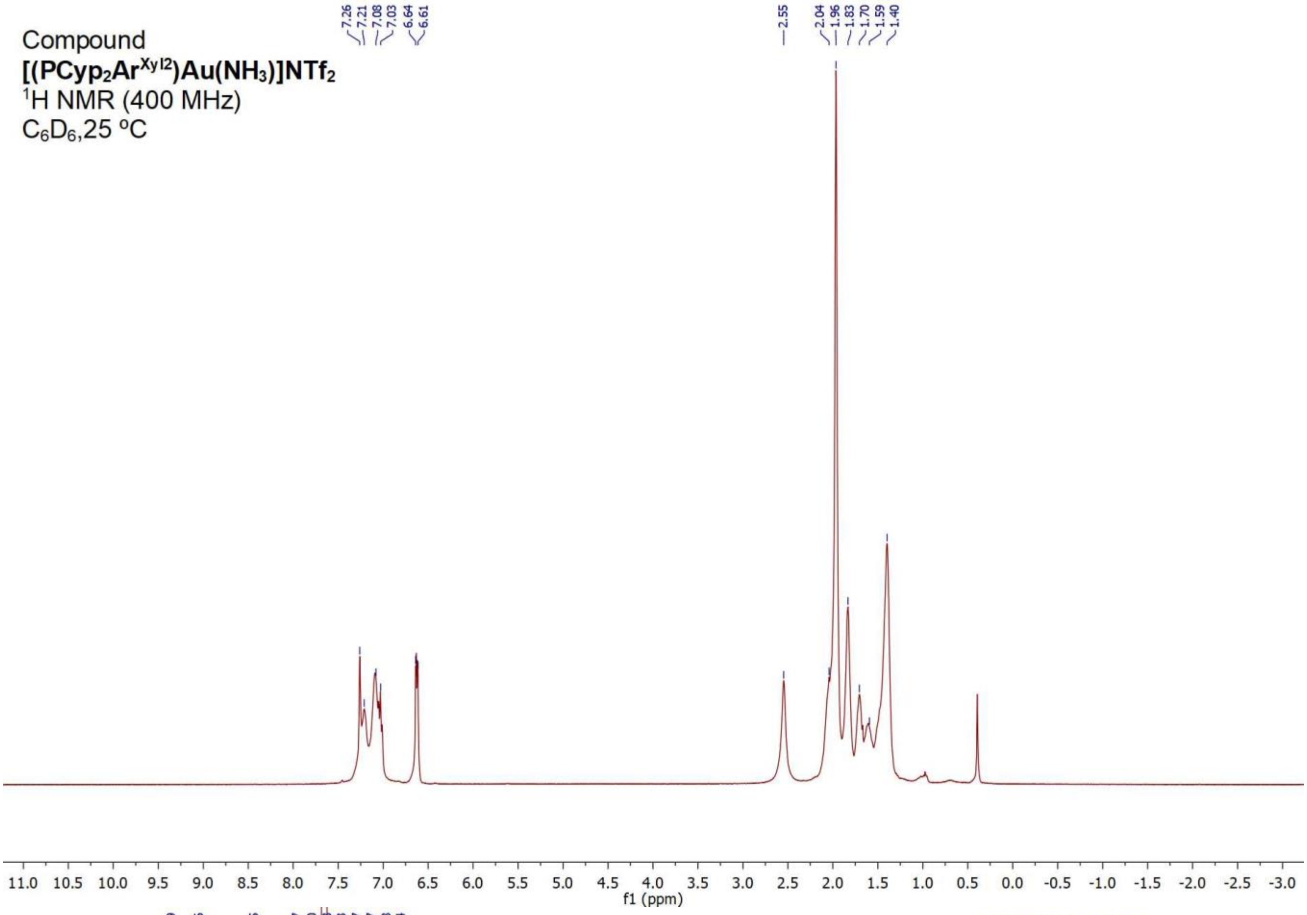
Compound
[(PCyp$_2$Ar^{Xyl2})Au(NH$_3$)]NTf$_2$
^{1}H NMR (400 MHz)
C_6D_6,25 ºC
7.26 7.21 7.08 7.03 6.64 6.61
2.55 2.04 1.96 1.83 1.70 1.59 1.40
11.0 10.5 10.0 9.5 9.0 8.5 8.0 7.5 7.0 6.5 6.0 5.5 5.0 4.5 4.0 3.5 3.0 2.5 2.0 1.5 1.0 0.5 0.0 -0.5 -1.0 -1.5 -2.0 -2.5 -3.0
f1 (ppm)

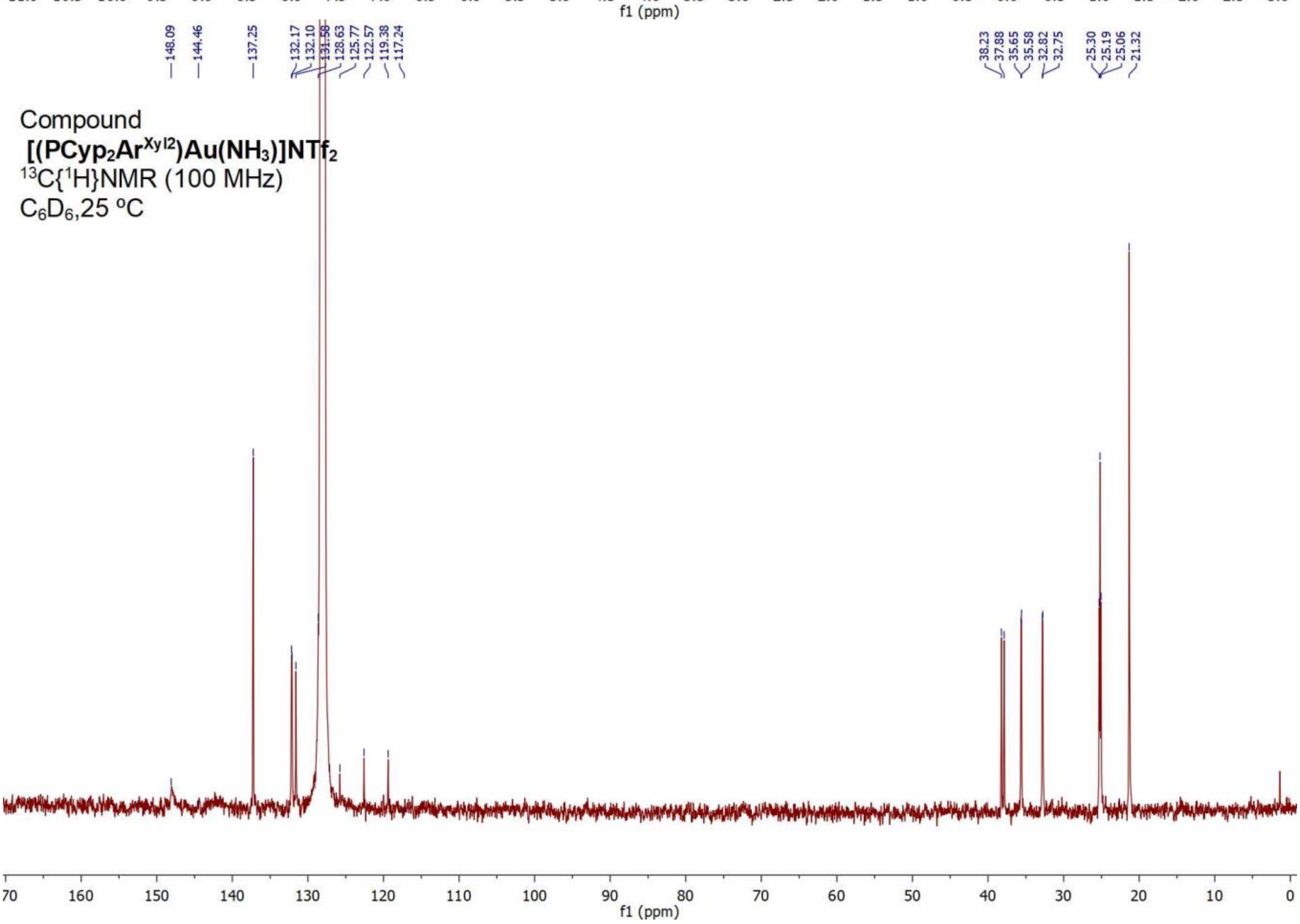
Compound
[(PCyp$_2$Ar^{Xyl2})Au(NH$_3$)]NTf$_2$
^{13}C{^{1}H}NMR (100 MHz)
C_6D_6,25 ºC
148.09 144.46 137.25 132.17 132.10 131.58 128.63 125.77 122.57 119.38 117.24
38.23 37.88 35.65 35.58 32.82 32.75 25.30 25.19 25.06 21.32
70 160 150 140 130 120 110 100 90 80 70 60 50 40 30 20 10 0
f1 (ppm)

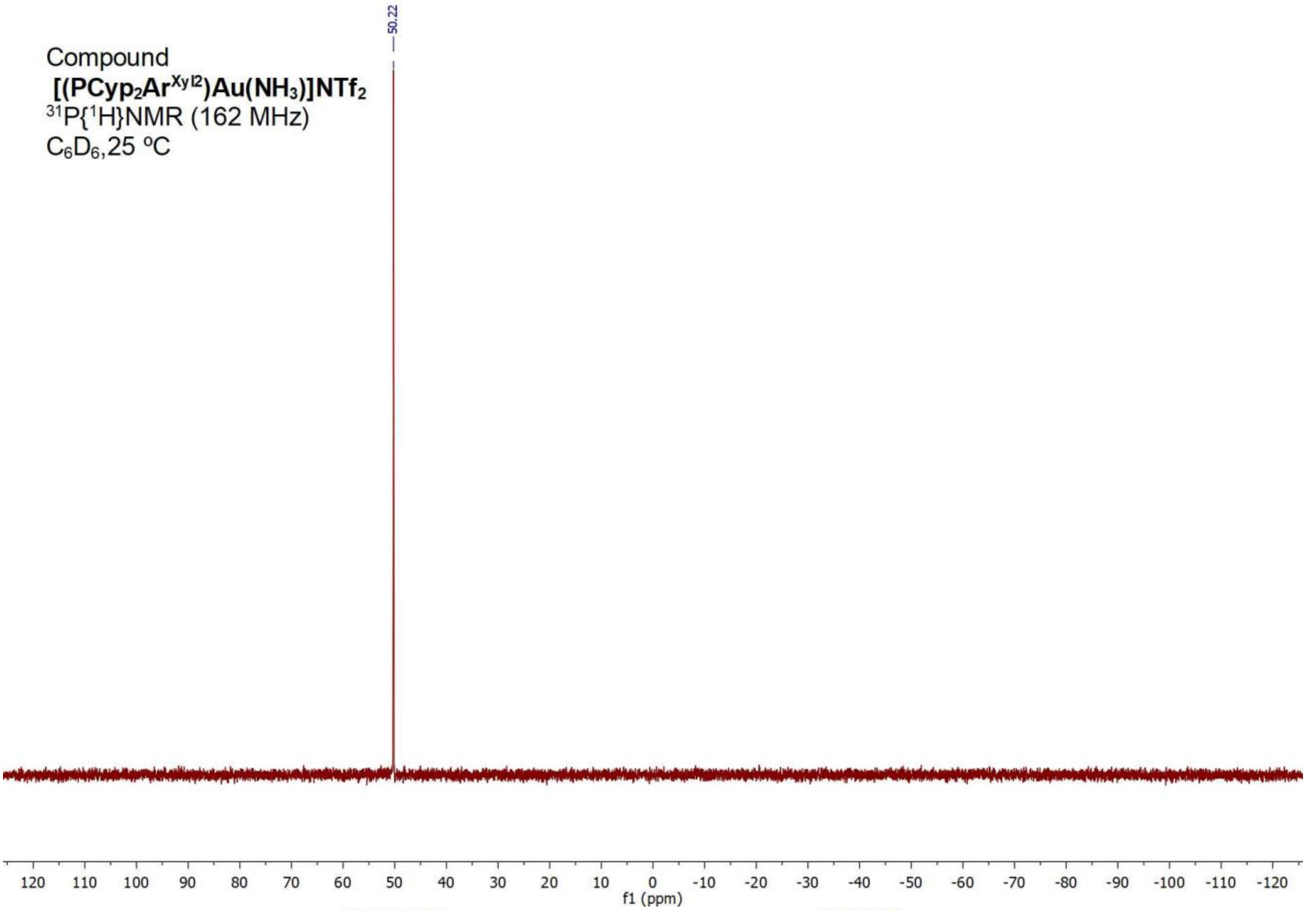
Compound
[(PCyp$_2$Ar^{Xyl2})Au(NH$_3$)]NTf$_2$
^{31}P{^{1}H}NMR (162 MHz)
C$_6$D$_6$,25 ºC
50.22
f1 (ppm)

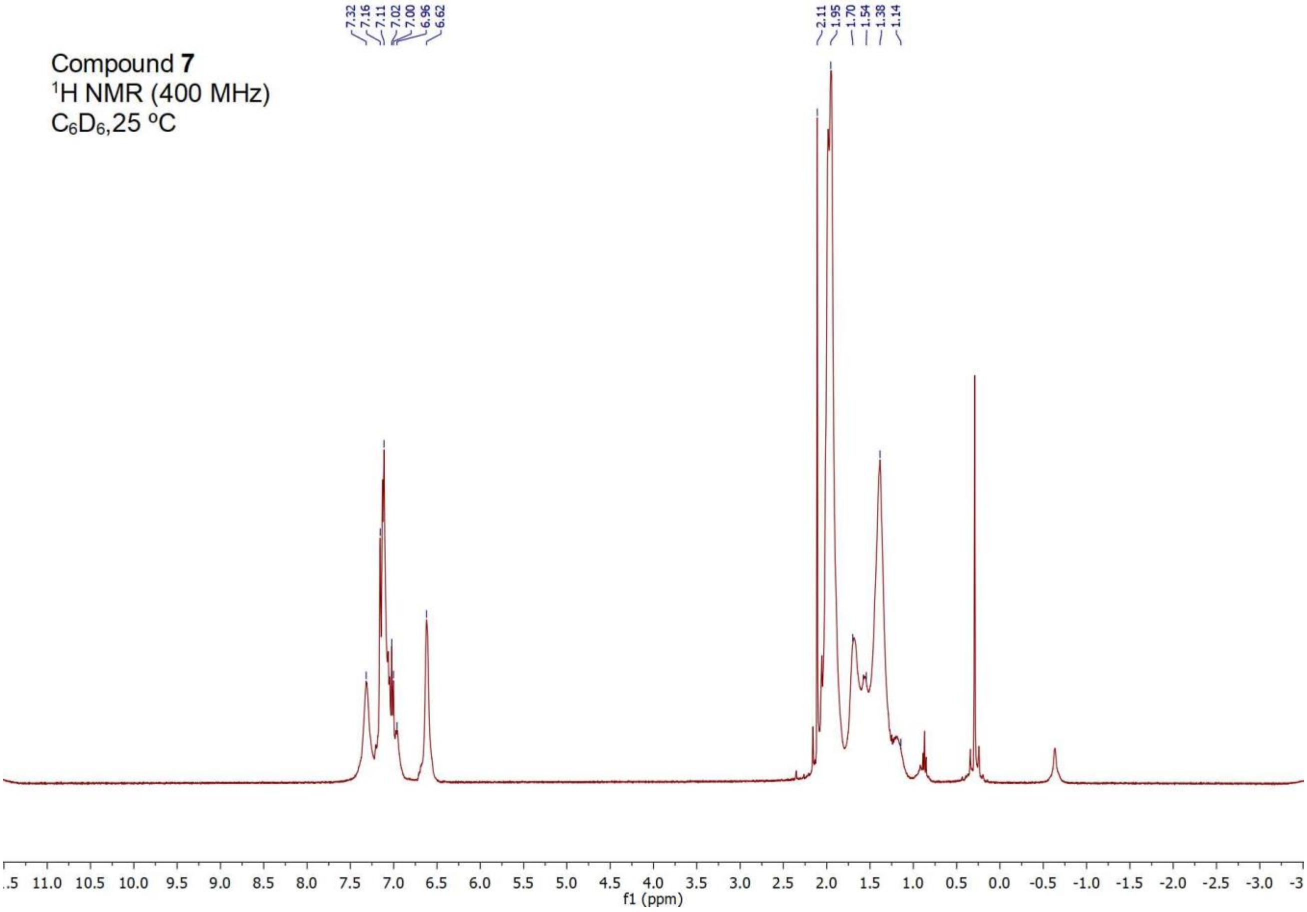
Compound 7
^{1}H NMR (400 MHz)
C$_6$D$_6$,25 ºC
7.32
7.16
7.11
7.02
7.00
6.96
6.62
2.11
1.95
1.70
1.54
1.38
1.14
f1 (ppm)

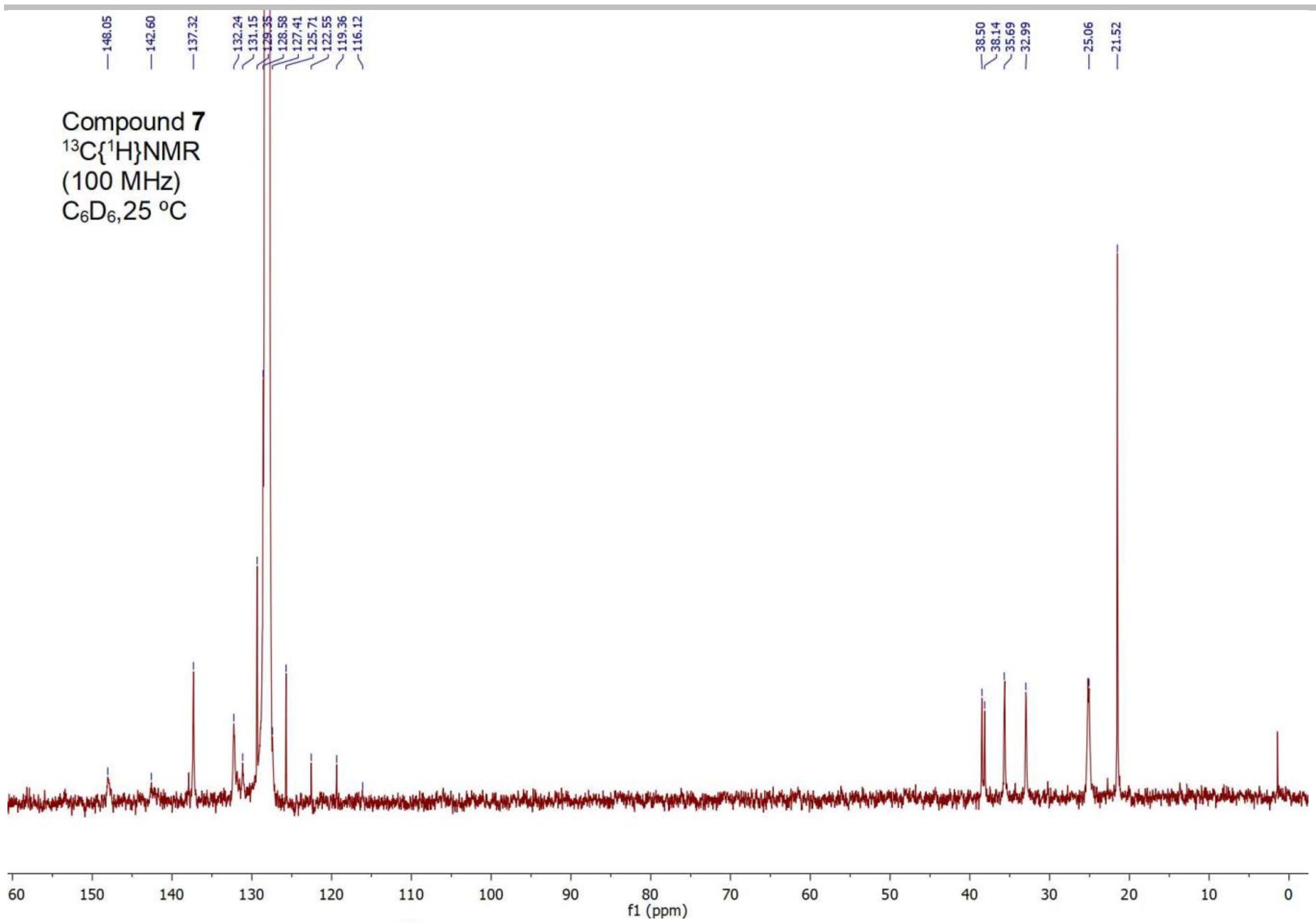
Compound 7
13C{1H}NMR
(100 MHz)
C6D6,25 ºC
148.05
142.60
137.32
132.24
131.15
129.35
128.58
127.41
125.71
122.55
119.36
116.12
38.50
38.14
35.69
32.99
25.06
21.52
f1 (ppm)

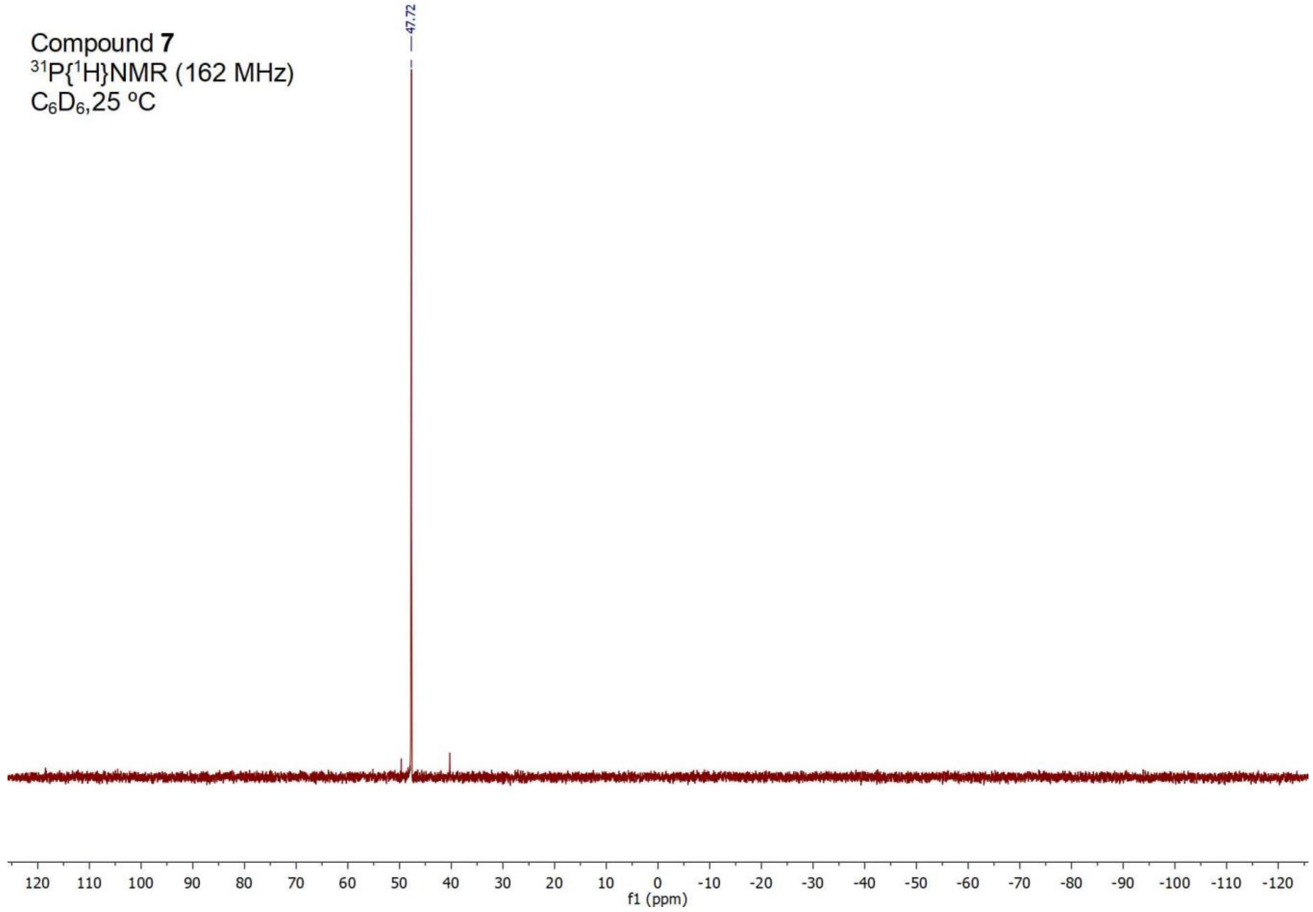
Compound 7
31P{1H}NMR (162 MHz)
C6D6,25 ºC
47.72
f1 (ppm)

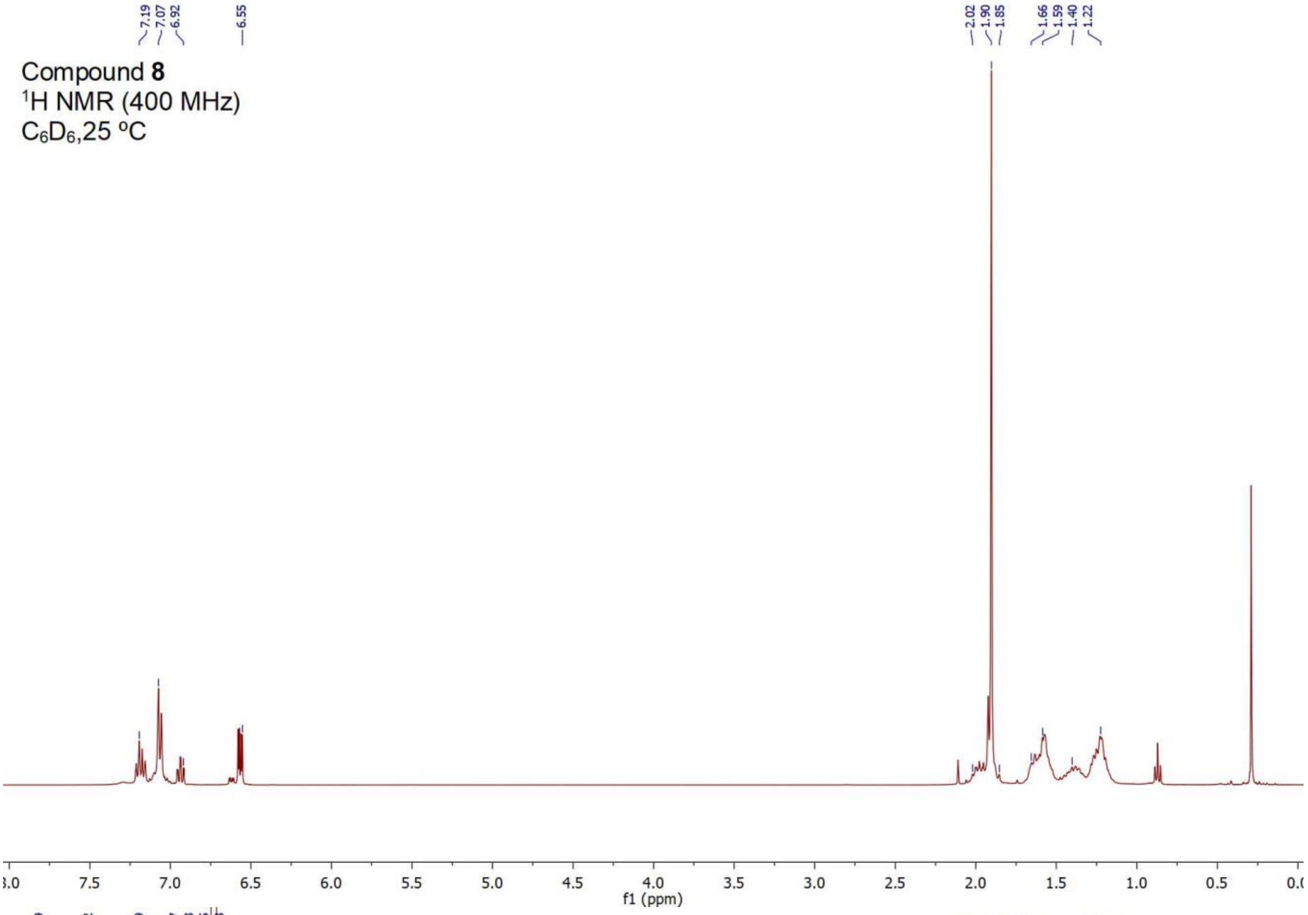
7.19
7.07
6.92
6.55
2.02
1.90
1.85
1.66
1.59
1.40
1.22
Compound **8**
^{1}H NMR (400 MHz)
C_6D_6,25 ºC
f1 (ppm)

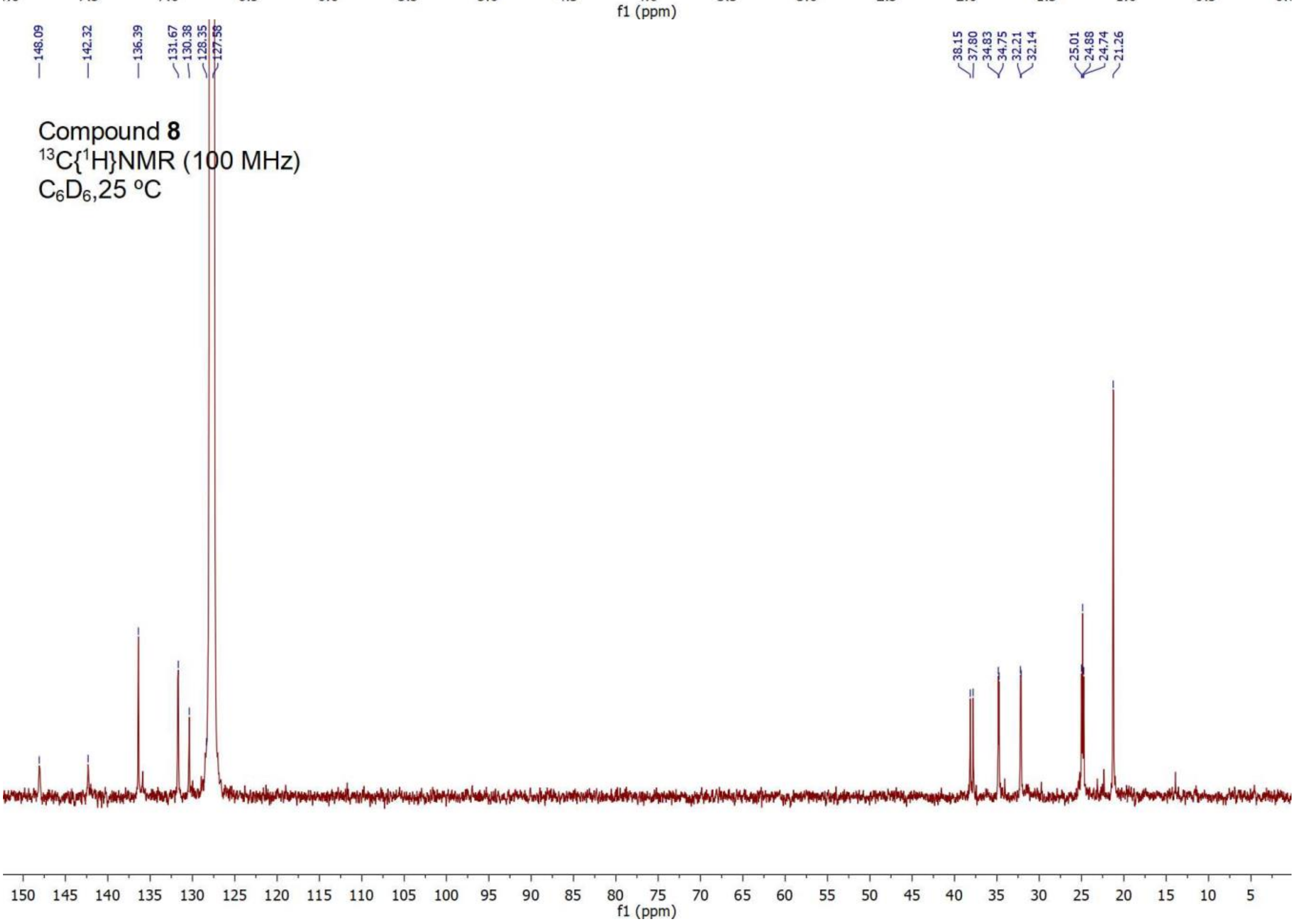
148.09
142.32
136.39
131.67
130.38
128.35
127.58
38.15
37.80
34.83
34.75
32.21
32.14
25.01
24.88
24.74
21.26
Compound **8**
^{13}C{^{1}H}NMR (100 MHz)
C_6D_6,25 ºC
f1 (ppm)

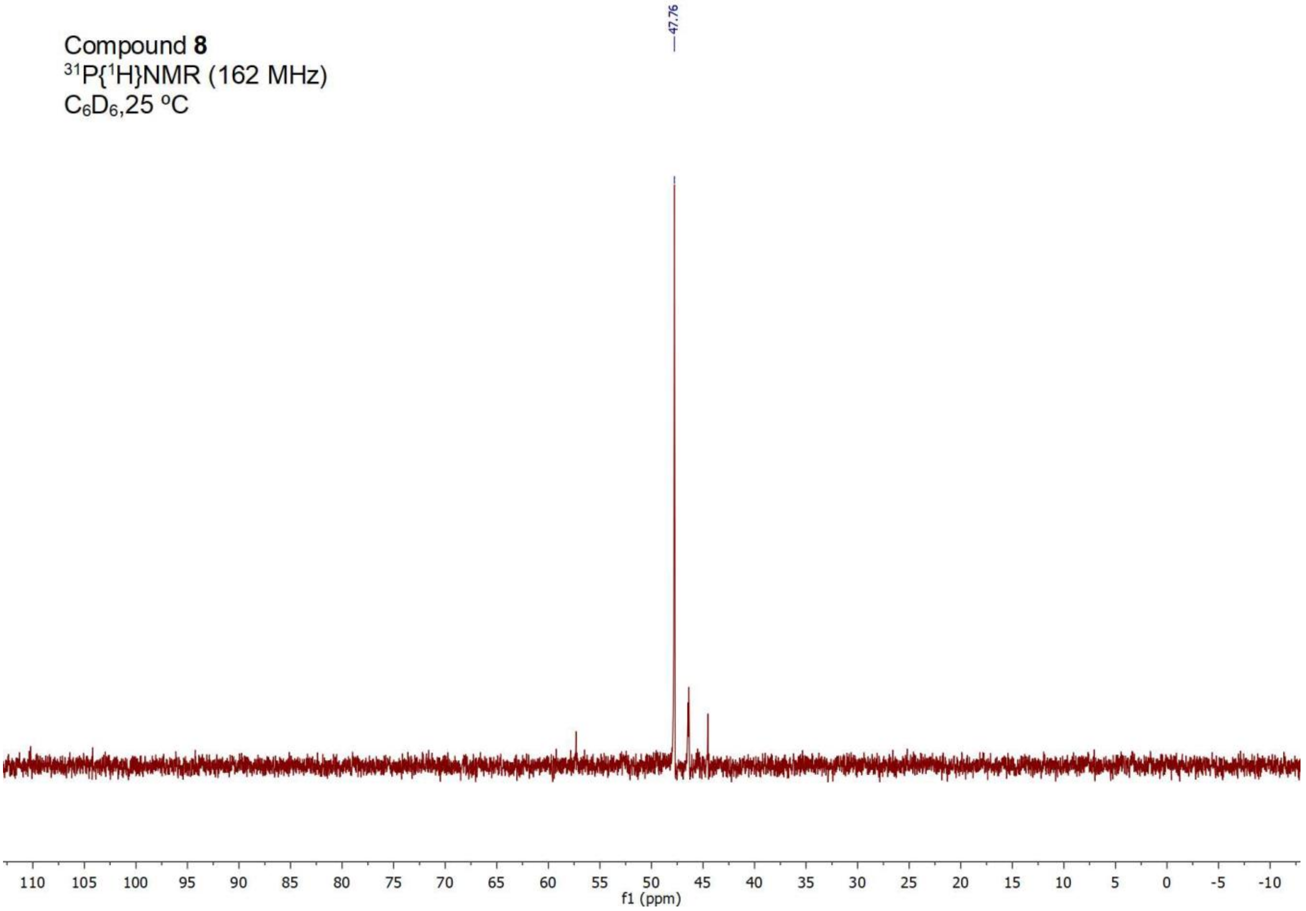

7. References

[1] A. Uson, M. Laguna, D. A. Briggs, H. H. Murray and J. P. Fackler, *Inorg. Synth*., **2007**, *26*, 85.
[2] H. Werner, B. Klingert, B. *Chem. Ber*. **1983**, *116*, 1450.
[3] J. Campos, M. F. Espada, , J. López-Serrano, M. L. Poveda, E. Carmona, *Angew. Chemie - Int. Ed.* **2015**, *54*, 15379.
[4] J. Campos, N. Hidalgo, J.J. Moreno, M. Pérez-Jiménez, C. Maya, J. López-Serrano, *Chem. Eur. J.* **2020,** *26* (27), 5982.
[5] SAINT 6.02*, BRUKER-AXS*, Inc., Madison, WI 53711-5373 USA, **1997–1999**.
[6] SADABS George Sheldrick, *Bruker AXS, Inc*., Madison,Wisconsin, USA, **1999**.
[7] G. M. Sheldrick, *Acta Cryst***. 2008**, A64, 112–122.
[8] M. J. Frisch, G. W. Trucks, H. B. Schlegel, G. E. Scuseria, M. A. Robb, J. R. Cheeseman, G. Scalmani, V. Barone, B. Mennucci, G. A. Petersson, H. Nakatsuji, M. Caricato, X. Li, H. P. Hratchian, A. F. Izmaylov, J. Bloino, G. Zheng, J. L. Sonnenberg, M. Hada, M. Ehara, K. Toyota, R. Fukuda, J. Hasegawa, M. Ishida, T. Nakajima, Y. Honda, O. Kitao, H. Nakai, T. Vreven, J. A. Montgomery, Jr., J. E. Peralta, F. Ogliaro, M. Bearpark, J. J. Heyd, E. Brothers, K. N. Kudin, V. N. Staroverov, T. Keith, R. Kobayashi, J. Normand, K. Raghavachari, A. Rendell, J. C. Burant, S. S. Iyengar, J. Tomasi, M. Cossi, N. Rega, J. M. Millam, M. Klene, J. E. Knox, J. B. Cross, V. Bakken, C. Adamo, J. Jaramillo, R. Gomperts, R. E. Stratmann, O. Yazyev, A. J. Austin, R. Cammi, C. Pomelli, J. W. Ochterski, R. L. Martin, K. Morokuma, V. G. Zakrzewski, G. A. Voth, P. Salvador, J. J. Dannenberg, S. Dapprich, A. D. Daniels, O. Farkas, J. B. Foresman, J. V. Ortiz, J. Cioslowski, and D. J. Fox, Gaussian, Inc., Wallingford CT, **2013**.
[9] Perdew, J. P.; Burke, K.; Ernzerhof, M. *Phys. Rev. Lett.* **1996**, *77*, 3865.
[10] Grimme, S.; Antony, J.; Ehrlich, S.; Krieg, H. *J. Chem. Phys*. **2010**, *132*, 154104.
[11] a) R. Ditchfield, W. J. Hehre, J. A. Pople. *Self-Consistent Molecular-Orbital Methods. IX. An Extended Gaussian-Type Basis for Molecular-Orbital Studies of Organic Molecules*. *J. Chem. Phys*. **1971**, *54*, 724–728; b) W. J. Hehre, R. Ditchfield, J. A. Pople. *Self—Consistent Molecular Orbital Methods. XII. Further Extensions of Gaussian-Type Basis Sets for Use in Molecular Orbital Studies of Organic Molecules*. *J. Chem. Phys*. **1972**, *56*, 2257–2261; c) P. C. Hariharan, J. A. Pople. *The Influence of Polarization Functions on Molecular Orbital Hydrogenation Energies*. *Theor. Chim. Acta* **1973**, *28*, 213–222; d) M. M. Francl, W. J. Pietro, W. J. Hehre, J. S. Binkley, M. S. Gordon, D. J. DeFrees, J. A. Pople. *Self-Consistent Molecular Orbital Methods. XXIII. A Polarization-Type Basis Set for Second-Row Elements*. *J. Chem. Phys*. **1982**, *77*, 3654–3665.
[12] D. Andrae, U. Haeussermann, M. Dolg, H. Stoll, H. Preuss. *Energy-Adjusted Ab Initio Pseudopotentials for the Second and Third Row Transition Elements*. *Theor. Chim. Acta* **1990**, *77*, 123–141.
[13] A. V. Marenich, C. J. Cramer, D. G. Truhlar. *Universal Solvation Model Based on Solute Electron Density and on a Continuum Model of the Solvent Defined by the Bulk Dielectric Constant and Atomic Surface Tensions*. *J. Phys. Chem. B* **2009**, *113*, 6378–6396.
[14] R. F. W. Bader. Atom in Molecules: A Quantum Theory; Oxford University Press: Oxford, UK, **1995**.
[15] a) T. Lu, F. Chen. *Multiwfn: A multifunctional wavefunction analyser*. *J. Comput. Chem*., **2012**, *33*, 580-592; b) Multiwfn 3.6, http://sobereva.com/multiwfn/
